# Clinical situations for which 3D printing is considered an appropriate representation or extension of data contained in a medical imaging examination: adult cardiac conditions

**DOI:** 10.1186/s41205-020-00078-1

**Published:** 2020-09-23

**Authors:** Arafat Ali, David H. Ballard, Waleed Althobaity, Andy Christensen, Mariah Geritano, Michelle Ho, Peter Liacouras, Jane Matsumoto, Jonathan Morris, Justin Ryan, Rami Shorti, Nicole Wake, Frank J. Rybicki, Adnan Sheikh, on behalf of the RSNA 3D Printing Special Interest Group

**Affiliations:** 1 Department of Radiology, University of Cincinnati Medical Center, Cincinnati, OH USA; 2 Mallinckrodt Institute of Radiology, Washington University School of Medicine, Saint Louis, MO USA; 3 King Faisal Specialist Hospital and Research Centre, Riyadh, Saudi Arabia; 4 Department of Radiology and The Ottawa Hospital Research Institute, University of Ottawa, Ottawa, ON Canada; 5 Boston Children’s Hospital, Boston, MA USA; 6 Sidney Kimmel Medical College, Thomas Jefferson University, Philadelphia, PA USA; 7 3D Medical Applications Center, Walter Reed National Military Medical Center, Washington, DC USA; 8 Department of Radiology, Mayo Clinic, Rochester, MN USA; 9 Rady Children’s Hospital, San Diego, CA USA; 10 Intermountain Healthcare, South Jordan, UT USA; 11 Department of Radiology, Montefiore Medical Center, Bronx, NY USA

**Keywords:** 3D printing, Appropriateness, Guidelines, Quality, Radiology, Additive Manufacturing, Anatomic Model, Adult Cardiology, Left Atrial Appendage, Transcatheter Aortic Valve Replacement

## Abstract

**Background:**

Medical 3D printing as a component of care for adults with cardiovascular diseases has expanded dramatically. A writing group composed of the Radiological Society of North America (RSNA) Special Interest Group on 3D Printing (SIG) provides appropriateness criteria for adult cardiac 3D printing indications.

**Methods:**

A structured literature search was conducted to identify all relevant articles using 3D printing technology associated with a number of adult cardiac indications, physiologic, and pathologic processes. Each study was vetted by the authors and graded according to published guidelines.

**Results:**

Evidence-based appropriateness guidelines are provided for the following areas in adult cardiac care; cardiac fundamentals, perioperative and intraoperative care, coronary disease and ischemic heart disease, complications of myocardial infarction, valve disease, cardiac arrhythmias, cardiac neoplasm, cardiac transplant and mechanical circulatory support, heart failure, preventative cardiology, cardiac and pericardial disease and cardiac trauma.

**Conclusions:**

Adoption of common clinical standards regarding appropriate use, information and material management, and quality control are needed to ensure the greatest possible clinical benefit from 3D printing. This consensus guideline document, created by the members of the RSNA 3D printing Special Interest Group, will provide a reference for clinical standards of 3D printing for adult cardiac indications.

## Background

In 2018, the RSNA 3D printing SIG published guidelines that include medical 3D printing appropriateness [1]. Those guidelines include 3D printing for patients with congenital heart disease. Medical 3D printing is performed for a variety of adult cardiac indications, but without evidence-based appropriate use criteria (AUC). The purpose of this document is to bridge the large unmet need to identify, vet, vote and publish appropriateness recommendations for 3D printing of adult cardiac indications.

## Methods

The Special Interest Group has initiated quality and safety scholarship to identify those clinical situations for which adult cardiac 3D Printing is considered usually appropriate, maybe appropriate and rarely appropriate as a representation of the data contained in a medical imaging examination. This document highlights appropriateness of adult cardiac 3D printing for clinical utilization, research, scientific, and informational purposes. This work is loosely modeled after the American College of Radiology Appropriateness Criteria® [2], in that the guidelines committee uses an evidence-based approach at scoring. Consensus among members is used when there is a paucity of evidence. Strength of evidence is determined by literature review.

The SIG Guidelines Chairperson oversees the ratings via a vote among Special Interest Group members who attend in-person meetings. The results of the ratings follow the following 1–9 format (with 9 being the most appropriate):

- 1–3, red, rarely appropriate: There is a lack of a clear benefit or experience that shows an advantage over usual practice.
- 4–6, yellow, maybe appropriate: There may be times when there is an advantage, but the data are lacking, or the benefits have not been fully defined.
- 7–9, green, usually appropriate: Data and experience shows an advantage to 3D printing as a method to represent and/or extend the value of data contained in the medical imaging examination.

Clinical scenarios were organized by two major cardiovascular treatises to (a) ensure an exhaustive, structured English language PubMed literature search (Additional file 1) performed October 2019, and (b) generate a document following the typical format of an Appropriate Use Criteria (AUC), including a structure using standard categories of cardiovascular disease [3, 4]. For each category, from the pool of total results, the number of publications considered “relevant results” was curated by consensus between physicians with expertise in 3D printing and cardiovascular care. 3D printing as an educational tool was not grouped in an individual category; instead, its value was considered with respect to individual clinical scenarios. Relevant publications which were not retrieved by the structured PubMed search were manually entered into the appropriate categories and indicated as such (Additional file 1). The following categories were excluded because they were considered outside the project scope (Virtual & augmented reality, Bioprinting, Molecular biology, Genetics, Molecular imaging, Diabetes, Endocrinology, and Thrombosis) or covered elsewhere. While this document addresses adult cardiac conditions, it does not address congenital heart disease in the adult. Cardiac 3D printing review articles were not considered in determining final appropriateness ratings [5–19]. All final components of this section were vetted and approved by vote of Special Interest Group members face-to-face at the 2019 Annual Meeting of the Radiological Society of North America (December 2, 2019, Chicago, IL, USA). In addition, all included studies [20–143] were graded with a strength of evidence assessment (Additional file 2) according to the ACR Appropriateness Criteria® Evidence Document [144].

## Results

This section provides evidence-based guidelines, supplemented by expert opinion when there is a paucity of peer-review data, to define and support the use of 3D printing for patients with adult cardiac disease [Table 1]. A total of 135 articles published between February 2007 and October 2019 were ultimately included in the evidence base (Additional file 1). The citations included in forming the appropriateness criteria and the strength of evidence assessment are presented in Additional files 1 and 2, respectively.

**Table 1:** Appropriateness Ratings for Adult Cardiac Indications

Clinical Condition	Rating	References
**Cardiac Fundamentals**		
Cardiovascular Pathology	**1**	-
Cardiovascular Physiology	**4**	[20–27]
Electrocardiography	**1**	-
Cardiac Resuscitation	**1**	-
**Perioperative and Intraoperative Care**		
Extracorporeal Circulation	**2**	[21]
**Coronary Disease and Ischemic Heart Disease**		
Coronary Artery Disease and Myocardial Infarction	**7**	[22–32]
Coronary Artery Fistula	**5**	[33–35]
Coronary Artery Aneurysm	**5**	[36]
Coronary Artery Bypass	**3**	-
Post-Surgical Infarction	**1**	-
Atherosclerosis	**4**	-
Chest pain, angina	**1**	-
**Complications of myocardial Infarction**		
Left ventricular aneurysm	**6**	-
Post infarct ventricular septal defect	**7**	[37]
Myocardial rupture, acute	**1**	-
Myocardial rupture, chronic	**4**	-
Left ventricle pseudoaneurysm	**5**	[38]
**Aortic Valve Disease**		
Transcatheter aortic valve replacement	**9**	[20, 39–59]
Surgical Aortic valve replacement	**5**	[60–62]
**Mitral Valve Disease**		
Transcatheter Mitral Valve Replacement	**9**	[63–81]
Surgical Mitral Valve Replacement	**7**	[82–89]
**Tricuspid Valve Disease**		
Tricuspid valve repair/replacement	**7**	[90–95]
**Pulmonic Valve Disease**		
Pulmonary valve repair/replacement	**7**	[96–98]
**Cardiac Arrhythmias**		
Cardiac Arrhythmia/atrial fibrillation	**6**	[99, 100]
Cardiac Pacing	**6**	[101, 102]
**Cardiac Neoplasm**		
Cardiac Tumors	**7**	[103–110]
**Cardiac Transplant and Mechanical Circulatory Support**		
Cardiac transplant	**7**	[111]
Left Ventricular Assist device	**7**	[112–114]
Total Artificial Heart	**3**	-
**Heart Failure**		
Heart Failure	**2**	-
**Preventative Cardiology**		
Blood pressure disorders (hypertension, hypotension)	**1**	-
Left atrial appendage occlusion	**9**	[115–132]
**Cardiac and Pericardial Disease**		
Hypertrophic Cardiomyopathy	**9**	[133–143]
Dilated and restrictive cardiomyopathy	**5**	-
Infectious and inflammatory conditions of the heart	**3**	-
Pericardial Disease	**4**	-
**Cardiac Trauma**		
Cardiac trauma	**1**	-

## Discussion

### Cardiac fundamentals

3D printed anatomic models provide a unique avenue for the study of complex hemodynamic function. Several research methods study cardiac hemodynamics using a 3D printed model [20–27], but none are applied clinically. No clinical applications exist on cardiac pathology or resuscitation.

### Perioperative and intraoperative care

A single study evaluated the feasibility of 3D printed models for evaluating flow dynamics extracorporeal circulation [21]; however, this study was not for direct use in patient care.

### Coronary disease and ischemic heart disease

Acute myocardial infarction is a life threatening cardiac emergency with significant associated morbidity and mortality [145]. Lead time for 3D segmentation and printing precludes the availability of anatomic models for real time procedural planning percutaneous intervention. As such, there are minimal results for the use of 3D printed models for myocardial infarction. Several authors assess the feasibility of 3D printed models for studying coronary arterial flow dynamics, but the results were not directly applied to patient care [22–27]. There are several published case reports of 3D printed models used in procedural planning for coronary artery aneurysm and fistula repair [33–36].

### Complications of myocardial infarction

Complications of myocardial infarction may require surgical management, as in the single case reports of a left ventricular pseudoaneurysm and post infarct ventricular septal defect, where a model was used for procedural planning [37, 38].

### Valve disease

Paravalvular leak is a potentially life-threatening complication of transcatheter valve replacement. Although no large-scale prospective studies have been performed, there is growing body of case reports, suggesting decreased incidence of paravalvular leak and improved outcomes for transcatheter aortic and mitral valve replacement planned with the use of a 3D printed anatomic model [20, 39–59, 63–81]. Complicated surgically placed aortic and mitral valve replacements, as well as pulmonary or tricuspid valves, may also benefit from anatomic models [60–62, 82–98].

### Cardiac arrhythmias

Irregular heart rhythms refractory to medical therapy may require cardiac ablation or pacemaker placement for definitive therapy. Published uses of anatomic models include case reports for procedural planning in cases with complex anatomy [99–102].

### Cardiac transplant and mechanical circulatory support

Cardiac transplantation is indicated in patients with end-stage heart failure who are symptomatic, despite optimization of medical therapy. Approximately 3200 transplantations are performed annually in the United States [146]. Anatomic models can potentially reduce operative time and outcomes for this complex surgical procedure. To date, there is only a single case report describing the use of a 3D printed model in an adult requiring a surgically challenging cardiac transplant with congenital heart disease [111].

Left ventricular assistive devices (LVAD) are a temporizing procedure for patients pending definite cardiac transplantation. An anatomic model was used to guide cannula placement and trabeculae resection in a single case report for LVAD placement [113].

### Heart failure

Heart failure portends a wide variety of causes and is usually managed with medical therapy preceding definitive cardiac transplantation. As such, no published articles describe the utility of 3D models for heart failure.

### Preventative cardiology

Patients with atrial fibrillation carry increased risk for stroke and a large meta-analysis of left atrial appendage closure demonstrates noninferiority compared with anticoagulation in patients with nonvalvular atrial fibrillation [147]. Growing evidence suggests superior outcomes with device sizing guided by an anatomic model [115–131].

There is no published evidence for the utility of anatomic models in the management of blood pressure disorders.

### Cardiac and pericardial disease

Accurate septal myectomy is the determinant of outcomes in surgical management of hypertrophic obstructive cardiomyopathy. Anatomic models can help to achieve superior intraventricular septum resection volume and shape [133–143].

Limited surgical options exist for dilated cardiomyopathy, restrictive cardiomyopathy, infectious or inflammatory conditions of the heart or pericardial disease. As such, there are no published data on the use of anatomic models in these conditions.

### Cardiac trauma

Blunt cardiac trauma may result in myocardial contusion with more severe blunt or penetrating trauma often resulting in exsanguination, pericardial tamponade or death. Treatable serious cardiac trauma requires resuscitation and emergent surgical management, thereby precluding the use of a 3D printed anatomic model due to the necessary segmentation and printing time.

## Conclusion

This document provides initial appropriateness recommendations for 3D printing in adult cardiac pathology. Ratings used available clinical evidence primarily from structured searches plus expert opinion when there is a paucity of evidence, recognizing that sparse data mandates that individual opinions can weigh heavily on appropriateness recommendations [148]. AUC as defined in the United States by the Centers of Medicare and Medicaid include multidisciplinary society input [149]. One limitation of the current SIG recommendations is that additional expert opinion, in this case from practicing cardiologists and cardiac surgeons, is lacking. This is an important gap, particularly as AUC and related documents move towards the peer-review literature [150, 151]. Adoption of common clinical standards regarding appropriate use, information and material management, and quality control are needed to ensure the greatest possible clinical benefit from 3D printing. It is anticipated that this consensus guideline document, created by the members of the RSNA 3D printing Special Interest Group, will provide a reference for clinical standards of 3D printing. The document will be periodically refined, based on expanding clinical applications and growing medical literature.

## Supplementary information

**Additional file 1:.** Supporting evidence obtained through structured PubMed searches. For each category, from the pool of total results, the number of publications considered “Relevant results” was curated by consensus between physicians with expertise in 3D printing and cardiovascular care. Relevant publications which were not retrieved by the structured PubMed search were manually entered into the appropriate categories and indicated accordingly.

**Additional file 2:.** Grading of each included study with a strength of evidence assessment according to ACR Appropriateness Criteria Evidence Document [144]. Studies were categorized as either primarily diagnostic (Dx), therapeutic (Tx), or both (Dx and Tx) along with a designation of observational, experimental, or review/other category. The review/other category is designated for studies that did not meet the definitions the ACR Evidence Document [144] for observational or experimental studies.

## Data Availability

The datasets used and/or analyzed during the current study are available from the corresponding author on reasonable request.

## Abbreviations

RSNA: Radiological Society of North America
SIG: Special Interest Group
AUC: Appropriate use criteria
LVAD: Left ventricular assistive device
