# Supplementary material for: Clinical situations for which 3D printing is considered an appropriate representation or extension of data contained in a medical imaging examination: adult cardiac conditions
Source: 3D Print Med. 2020 Sep 23;6:24. doi: 10.1186/s41205-020-00078-1 (PMC7510265; doi:10.1186/s41205-020-00078-1)
Supplement: Supplementary file 1 — Additional file 1:. Supporting evidence obtained through structured PubMed searches. For each category, from the pool of total results, the number of publications considered “Relevant results” was curated by consensus between physicians with expertise in 3D printing and cardiovascular care. Relevant publications which were not retrieved by the structured PubMed search were manually entered into the appropriate categories and indicated accordingly. [file 41205_2020_78_MOESM1_ESM.docx]

**Appendix 1.** Supporting evidence obtained through structured PubMed searches. For each category, from the pool of total results, the number of publications considered “Relevant results” was curated by consensus between physicians with expertise in 3D printing and cardiovascular care. Relevant publications which were not retrieved by the structured PubMed search were manually entered into the appropriate categories and indicated accordingly.

**Cardiac Fundamentals (retrieved October 2019)**

Cardiovascular Pathology

PubMed Search: ((3D printing) AND (cardiovascular pathology)) OR ((rapid prototyping) AND (cardiovascular pathology))

Total Results: [N=60]

Relevant results: None

Cardiovascular Physiology

PubMed Search: ((3D printing) AND (cardiovascular physiology)) OR ((rapid prototyping) AND (cardiovascular physiology))

Total Results: [N=212]

Relevant results: None

Manually entered publications: [N=8] ^20-27^

Electrocardiography

PubMed Search: ((3D printing) AND (Electrocardiogram)) OR ((rapid prototyping) AND (Electrocardiogram)) OR ((3D printing) AND (EKG)) OR ((rapid prototyping) AND (EKG))

Total Results: [N=11]

Relevant results: None

Cardiac Resuscitation

PubMed Search: ((3D printing) AND (cardiac resuscitation)) OR ((rapid prototyping) AND (cardiac resuscitation))

Total Results: [N=1]

Relevant results: None

**Table 1. Cardiac Fundamentals**

|  | **Article category** | **Number of articles** | **Number of patients** | **Used in clinical care** |
| --- | --- | --- | --- | --- |
| **Cardiovascular Pathology** | No relevant articles from this search query | | | |
| **Cardiovascular Physiology** | Training/Simulation | 1 | NA | No |
|  | Other/Feasibility | 7 | NA | No |
| **Electrocardiography** | No relevant articles from this search query | | | |
| **Cardiac Resuscitation** | No relevant articles from this search query | | | |

**Perioperative and Intraoperative Care (retrieved October 2019)**

Extracorporeal Circulation

PubMed Search: ((3D printing) AND (ECMO)) OR ((rapid prototyping) AND (ECMO)) OR ((3D printing) AND (extracorporeal circulation)) OR ((rapid prototyping) AND (extracorporeal circulation))

Total Results: [N=12]

Relevant results: [N=1]^21^

**Table 2. Extracorporeal Circulation**

|  | **Article category** | **Number of articles** | **Number of patients** | **Used in clinical care** |
| --- | --- | --- | --- | --- |
| **Extracorporeal Circulation** | Training/simulation | 1 | NA | No |

**Coronary Disease and Ischemic Heart Disease (retrieved October 2019)**

Coronary Disease and Ischemic Heart Disease

PubMed Search: ((3D printing) AND (coronary artery disease)) OR ((rapid prototyping) AND (coronary artery disease)) OR ((3D printing) AND (myocardial revascularization)) OR ((rapid prototyping) AND (myocardial revascularization)) OR ((3D printing) AND (myocardial infarction)) OR ((rapid prototyping) AND (myocardial infarction)) OR ((3D printing) AND (PCI)) OR ((rapid prototyping) AND (PCI)) OR ((3D printing) AND (coronary arteriography)) OR ((rapid prototyping) AND (coronary arteriography)) OR ((3D printing) AND (coronary angiography)) OR ((rapid prototyping) AND (coronary angiography))

Total Results: [N=43]

Relevant results: [N=15] **^22-36^**

Coronary Artery Bypass

PubMed Search: ((3D printing) AND (coronary artery bypass)) OR ((rapid prototyping) AND (coronary artery bypass)) OR ((3D printing) AND (CABG)) OR ((rapid prototyping) AND (CABG))

Total Results: [N=4]

Relevant Results: [N=0]

Post-Surgical Infarction

PubMed Search: ((3D printing) AND (Post-surgical infarction)) OR ((rapid prototyping) AND (Post-surgical infarction))

Total Results: [N=10]

Relevant Results: [N=0]

Complications of Myocardial Infarction

PubMed Search: ((3D printing) AND (left ventricle aneurysm)) OR ((rapid prototyping) AND (left ventricle aneurysm)) OR ((3D printing) AND (post infarct ventricular septal defect)) OR ((rapid prototyping) AND (post infarct ventricular septal defect)) OR ((3D printing) AND (myocardial rupture)) OR ((rapid prototyping) AND (myocardial rupture)) OR ((3D printing) AND (left ventricle pseudoaneurysm)) OR ((rapid prototyping) AND (left ventricle pseudoaneurysm)) OR ((3D printing) AND (post infarct ventricular septal defect)) OR ((3D printing) AND (ventricular septum)) OR ((rapid prototyping) AND (ventricular septum)) OR ((3D printing) AND (VSD)) OR ((rapid prototyping) AND (VSD))

Total Results: [N= 5]

Relevant Results: [N=2] [^37,38^]

Atherosclerosis

PubMed Search: ((3D printing) AND (atherosclerosis)) OR ((rapid prototyping) AND (atherosclerosis))

Total Results: [N=7]

Relevant Results: [N=0]

Chest Pain

PubMed Search: ((3D printing) AND (chest pain)) OR ((rapid prototyping) AND (chest pain)) OR ((3D printing) AND (angina)) OR ((rapid prototyping) AND (angina))

Total Results: [N=4]

Relevant Results: [N=0]

**Table 3. Atherosclerotic Cardiovascular Disease and Ischemic Heart Disease**

|  | **Article category** | **Number of articles** | **Number of patients** | **Used in clinical care** |
| --- | --- | --- | --- | --- |
| **Coronary Artery Disease and Myocardial Infarction** | Case Report | 2 |  | Yes |
|  | Other/Feasibility | 9 | NA | No |
| **Coronary Artery Fistula** | Case Report | 2 | 2 | Yes |
|  | Case Series | 1 | 4 | Yes |
| **Coronary Artery Aneurysm** | Case Report | 1 | 1 | Yes |
| **Coronary Artery Bypass** | No relevant articles from this search query | | | |
| **Post-surgical Infarction** | No relevant articles from this search query | | | |
| **Left ventricle pseudoaneurysm** | Case Report | 1 | 1 | Yes |
| **Postinfarct ventricular septal defect** | Case Report | 1 | 1 | Yes |
| **Atherosclerosis** | No relevant articles from this search query | | | |
| **Chest Pain** | No relevant articles from this search query | | | |

**Aortic Valve Disease (retrieved October 2019)**

Aortic Valve replacement

PubMed Search: ((3D printing) AND (aortic valve)) OR ((rapid prototyping) AND (aortic valve)) OR ((3D printing) AND (TAVR)) OR ((rapid prototyping) AND (TAVR)) OR ((3D printing) AND (aortic regurgitation)) OR ((rapid prototyping) AND (aortic regurgitation)) OR ((3D printing) AND (aortic stenosis)) OR ((rapid prototyping) AND (aortic stenosis)) OR ((3D printing) AND (transcatheter aortic valve replacement)) OR ((rapid prototyping) AND (transcatheter aortic valve replacement))

Total Results: [N=79]

Relevant Results [N=25]**^20,39-62^**

**Table 4. Aortic Valve Disease**

|  | **Article category** | **Number of articles** | **Number of patients** | **Used in clinical care** |
| --- | --- | --- | --- | --- |
| **Transcatheter Aortic Valve Replacement** | Case Report | 6 | 5 | Yes -5; No - 1 |
|  | Case Series | 2 | 9 | Yes – 1; Unclear – 1 |
|  | Other/Feasibility | 11 | NA | No |
|  | Training/Simulation | 1 | NA | No |
|  | Review | 2 | NA | No |
| **Surgical Aortic Valve Replacement** | Case Report | 3 | 3 | Yes |

**Mitral Valve Disease (retrieved October 2019)**

Mitral Valve repair/replacement

PubMed Search: ((3D printing) AND (mitral valve)) OR ((rapid prototyping) AND (mitral valve)) OR ((3D printing) AND (TMVR)) OR ((rapid prototyping) AND (TMVR)) OR ((3D printing) AND (mitral)) OR ((rapid prototyping) AND (mitral))

Total Results: [N=53]

Relevant Results: [N=28]^63-89^

**Table 5. Mitral Valve Disease**

|  | **Article category** | **Number of articles** | **Number of patients** | **Used in clinical care** |
| --- | --- | --- | --- | --- |
| **Transcatheter Mitral Valve Replacement** | Case Report | 4 | 4 | Yes – 4 |
|  | Case Series | 3 | 17 | Yes -2; Unclear -1 |
|  | Other/Feasibility | 10 | NA | No |
|  | Training/Simulation | 2 | NA | No |
|  | Review | 1 | NA | No |
| **Surgical Mitral Valve Replacement/Repair** | Case Report | 3 | 3 | Yes |
|  | Other/Feasibility | 4 | NA | No |
|  | Training/Simulation | 1 | NA | No |

**Tricuspid Valve Disease (retrieved October 2019)**

Tricuspid valve repair/replacement

PubMed Search: ((3D printing) AND (tricuspid valve)) OR ((rapid prototyping) AND (tricuspid valve)) OR ((3D printing) AND (caval valve)) OR ((rapid prototyping) AND (caval valve))

Total Results: [N=12]

Relevant Results: [N=6]^90-95^

**Table 6. Tricuspid Valve Disease**

|  | **Article category** | **Number of articles** | **Number of patients** | **Used in clinical care** |
| --- | --- | --- | --- | --- |
| **Tricuspid Valve Repair/Replacement** | Case Report | 1 | 1 | Yes |
|  | Case Series | 3 | 8 | Yes |
|  | Other/Feasibility | 1 | NA | No |
|  | Review | 1 | NA | No |

**Pulmonary Valve Disease (retrieved October 2019)**

Pulmonary valve repair/replacement

PubMed Search: ((3D printing) AND (pulmonary valve)) OR ((rapid prototyping) AND (pulmonary valve))

Total Results: [N=4]

Relevant results: [N=3]^96-98^

**Table 7. Pulmonary Valve Disease**

|  | **Article category** | **Number of articles** | **Number of patients** | **Used in clinical care** |
| --- | --- | --- | --- | --- |
| **Pulmonary Valve Repair/Replacement** | Case Report | 1 | 1 | Yes |
|  | Other | 2 | NA | No |

**Cardiac Arrhythmias (retrieved October 2019)**

Cardiac arrhythmia

PubMed Search: ((3D printing) AND (arrhythmia)) OR ((rapid prototyping) AND (arrhythmia)) OR ((3D printing) AND (atrial fibrillation)) OR ((rapid prototyping) AND (atrial fibrillation))

Total Results: [N=12]

Relevant Results: [N=2]^99,100^

Cardiac Pacing

PubMed Search: ((3D printing) AND (pacemaker)) OR ((rapid prototyping) AND (pacemaker)) OR ((3D printing) AND (defibrillation)) OR ((rapid prototyping) AND (defibrillation))

Total Results: [N=10]

Relevant Results: [N=2]^101,102^

**Table 8. Cardiac Arrhythmias**

|  | **Article category** | **Number of articles** | **Number of patients** | **Used in clinical care** |
| --- | --- | --- | --- | --- |
| **Cardiac Arrhythmias** | Case Report | 2 | 2 | Yes |
| **Cardiac Pacing** | Case Report | 2 | 2 | 1 – Yes; 1 – No |

**Cardiac Neoplasm (retrieved October 2019)**

Cardiac Tumors

PubMed Search: ((3D printing) AND (cardiac tumor)) OR ((rapid prototyping) AND (cardiac tumor)) OR ((3D printing) AND (cardiac neoplasm)) OR ((rapid prototyping) AND (cardiac neoplasm))

Total Results: [N=12]

Relevant Results: [N=8]^103-110^

**Table 9. Cardiac Neoplasm**

|  | **Article category** | **Number of articles** | **Number of patients** | **Used in clinical care** |
| --- | --- | --- | --- | --- |
| **Cardiac Tumors** | Case Report | 4 | 4 | Yes |
|  | Case Series | 2 | 4 | Yes |
|  | Review | 2 | NA | No |

**Cardiac Transplant and Mechanical Circulatory Support (retrieved October 2019)**

Cardiac Transplant

PubMed Search: ((3D printing) AND (cardiac transplant)) OR ((rapid prototyping) AND (cardiac transplant)) OR ((3D printing) AND (heart transplant)) OR ((rapid prototyping) AND (heart transplant))

Total Results: [N=32]

Relevant Results: [N=3]^111^

Left Ventricular Assist Device

PubMed Search: ((3D printing) AND (ventricular assist device)) OR ((rapid prototyping) AND (ventricular assist device)) OR ((3D printing) AND (LVAD)) OR ((rapid prototyping) AND (LVAD))

Total Results: [N=15]

Relevant Results: [N=3]^112-114^

Total Artificial Heart

PubMed Search: ((3D printing) AND (total artificial heart)) OR ((rapid prototyping) AND (total artificial heart)) or ((3D printing) AND (TAH)) OR ((rapid prototyping) AND (TAH))

Total Results: [N=3]

Relevant Results: [N=0]

**Table 10. Cardiac Transplant and Mechanical Circulatory Support**

|  | **Article category** | **Number of articles** | **Number of patients** | **Used in clinical care** |
| --- | --- | --- | --- | --- |
| **Cardiac Transplant** | Case Report | 2 | 2 | Yes |
|  | Case Series | 1 | 2 | Yes |
| **Left Ventricular Assist Device** | Case Report | 1 | 1 | Yes |
|  | Review | 2 | NA | No |
| **Total Artificial Heart** | No relevant articles from this search query | | | |

**Heart Failure (retrieved October 2019)**

Heart failure

PubMed Search: ((3D printing) AND (heart failure)) OR ((rapid prototyping) AND (heart failure)) OR ((3D printing) AND (cardiac failure)) OR ((rapid prototyping) AND (cardiac failure)) OR ((3D printing) AND (end stage heart disease)) OR ((rapid prototyping) AND (end stage heart disease)) OR ((3D printing) AND (end stage cardiac disease)) OR ((rapid prototyping) AND (end stage cardiac disease))

Total Results: [N=32]

Relevant Results: [N=0]

**Table 11. Heart Failure**

|  | **Article category** | **Number of articles** | **Number of patients** | **Used in clinical care** |
| --- | --- | --- | --- | --- |
| **Heart Failure** | No relevant articles from this search query | | | |

**Preventative Cardiology (retrieved October 2019)**

Blood pressure disorders

PubMed Search: ((3D printing) AND (hypertension)) OR ((rapid prototyping) AND (hypertension)) OR ((3D printing) AND (hypotension)) OR ((rapid prototyping) AND (hypotension))

Total Results: [N=17]

Relevant Results: [N=0]

Left Atrial Appendage Occlusion

PubMed Search: ((3D printing) AND (left atrial appendage)) OR ((rapid prototyping) AND (left atrial appendage))

Total Results: [N=31]

Relevant Results: [N=18] ^115-132^

**Table 12. Preventative Cardiology**

|  | **Article category** | **Number of articles** | **Number of patients** | | **Used in clinical care** |
| --- | --- | --- | --- | --- | --- |
| **Blood Pressure Disorder** | No relevant articles from this search query | | | | |
| **Left Atrial Appendage Exclusion** | Case Report | 3 | 3 | Yes | |
|  | Case Series | 3 | 12 | Yes | |
|  | Review | 2 | NA | No | |
|  | Prospective Cohort | 5 | 91 | Yes | |
|  | Retrospective Cohort | 4 | 149 | No | |
|  | Prospective and Retrospective Cohort | 1 | 104 | Yes | |

**Cardiac and Pericardial Disease (retrieved October 2019)**

Hypertrophic Cardiomyopathy

PubMed Search: ((3D printing) AND (myectomy)) OR ((rapid prototyping) AND (myectomy)) OR ((3D printing) AND (cardiomyopathy)) OR ((rapid prototyping) AND (cardiomyopathy))

Total Results: [N=14]

Relevant Results: [N= 11]^133-143^

Dilated and Restrictive Cardiomyopathy

PubMed Search: ((3D printing) AND (dilated cardiomyopathy)) OR ((rapid prototyping) AND (dilated cardiomyopathy)) OR ((3D printing) AND (restrictive cardiomyopathy)) OR ((rapid prototyping) AND (restrictive cardiomyopathy))

Total Results: [N=0]

Relevant Results: [N=0]

Infectious and Inflammatory Conditions of the Heart

PubMed Search: ((3D printing) AND (endocarditis)) OR ((rapid prototyping) AND (endocarditis)) OR ((3D printing) AND (myocarditis)) OR ((rapid prototyping) AND (myocarditis)) OR ((3D printing) AND (Chagas)) OR ((rapid prototyping) AND (Chagas))

Total Results: [N=6]

Relevant Results: [N=0]

Pericardial Disease

PubMed Search: ((3D printing) AND (pericardium)) OR ((rapid prototyping) AND (pericardium)) OR ((3D printing) AND (pericardial disease)) OR ((rapid prototyping) AND (pericardial disease))

Total Results: [N=9]

Relevant Results: [N=0]

**Table 13. Cardiac and Pericardial Disease**

|  | **Article category** | **Number of articles** | **Number of patients** | **Used in clinical care** |
| --- | --- | --- | --- | --- |
| **Hypertrophic Cardiomyopathy** | Case Report | 4 | 4 | Yes – 3; No - 1 |
|  | Case Series | 4 | 16 | Yes – 3; No - 1 |
|  | Review | 1 | NA | No |
|  | Prospective Cohort | 1 | 30 | Yes |
|  | Training/Simulation | 1 | NA | No |
| **Dilated and Restrictive Cardiomyopathy** | No relevant articles from this search query | | | |
| **Infectious and Inflammatory conditions of the heart** | No relevant articles from this search query | | | |
| **Pericardial Disease** | No relevant articles from this search query | | | |

**Cardiac Trauma**

Cardiac Trauma

PubMed Search: ((3D printing) AND (cardiac trauma)) OR ((rapid prototyping) AND (cardiac trauma))

Total Results: [N=14]

Relevant Results: [N=0]

**Table 14. Cardiac Trauma**

|  | **Article category** | **Number of articles** | **Number of patients** | **Used in clinical care** |
| --- | --- | --- | --- | --- |
| **Cardiac Trauma** | No relevant articles from this search query | | | |

**Review Articles [N=15] ^5-19^**
