# Supplementary material for: Clinical situations for which 3D printing is considered an appropriate representation or extension of data contained in a medical imaging examination: adult cardiac conditions
Source: 3D Print Med. 2020 Sep 23;6:24. doi: 10.1186/s41205-020-00078-1 (PMC7510265; doi:10.1186/s41205-020-00078-1)
Supplement: Supplementary file 2 — Additional file 2:. Grading of each included study with a strength of evidence assessment according to ACR Appropriateness Criteria Evidence Document [144]. Studies were categorized as either primarily diagnostic (Dx), therapeutic (Tx), or both (Dx and Tx) along with a designation of observational, experimental, or review/other category. The review/other category is designated for studies that did not meet the definitions the ACR Evidence Document [144] for observational or experimental studies. [file 41205_2020_78_MOESM2_ESM.docx]

**Appendix 2**. Grading of each included study with a strength of evidence assessment according to ACR Appropriateness Criteria Evidence Document.^144^ Studies were categorized as either primarily diagnostic (Dx), therapeutic (Tx), or both (Dx and Tx) along with a designation of observational, experimental, or review/other category. The review/other category is designated for studies that did not meet the definitions the ACR Evidence Document^144^ for observational or experimental studies.

| **Reference** | | **Study Type** | **Patients/Events** | **Study Objective (Purpose of Study)** | **Study Results** | **Study Quality** |
| --- | --- | --- | --- | --- | --- | --- |
| 20 | Paulsen MJ, Kasinpila P, Imbrie-Moore AM, et al. Modeling conduit choice for valve-sparing aortic root replacement on biomechanics with a 3-dimensional-printed heart simulator. J Thorac Cardiovasc Surg. 2019;158(2):392-403. | Review/Other-Dx | N/A | We hypothesized that straight tubular grafts may help maintain the native cylindrical position of the aortic valve commissures radially, resulting in preserved leaflet coaptation, reduced stresses, and potentially improved valve performance. | Hemodynamic parameters and coronary blood flow were similar between straight and Valsalva grafts, although the former were associated with lower regurgitant fractions, less peak intercommissural radial separation, preserved leaflet coaptation, decreased leaflet velocities, and lower relative leaflet forces compared with Valsalva grafts. | 4 |
| 21 | Ahn CB, Lee SI, Choi CH, et al. Feasibility of a 3D Printed Patient-Specific Model System to Determine Hemodynamic Energy Delivery During Extracorporeal Circulation. ASAIO J. 2018;64(3):309-317. | Review/Other-Dx | N/A | The authors constructed and tested the feasibility of a three-dimensional (3D) printed, patient-specific, silicone aortic model to determine whether aortic cannula tip positional changes affect energy equivalent pressure (EEP) and surplus hemodynamic energy (SHE) in carotid arteries. | Using this 3D printed silicone model of the ascending aorta, it was found that EEP and SHE of both right and left carotid arteries were significantly affected by aortic cannula tip position. | 4 |
| 22 | Modi BN, Ryan M, Chattersingh A, et al. Optimal Application of Fractional Flow Reserve to Assess Serial Coronary Artery Disease: A 3D-Printed Experimental Study With Clinical Validation. J Am Heart Assoc. 2018;7(20):e010279. | Observational-Dx | 30 | We aimed to use 3-dimensional-printing to characterize serial stenosis interplay and to derive and validate a mathematical solution to predict true stenosis significance in serial disease. | Δ FFR app underestimated Δ FFR true in 88% of phantoms, with underestimation proportional to total FFR . Discrepancy as a proportion of Δ FFR true was 17.1% (absolute difference 0.036±0.048), which improved to 2.9% (0.006±0.023) using our model. In the clinical cohort, discrepancy was 38.5% (0.05±0.04) with 13.3% of stenoses misclassified (using FFR <0.8 threshold). Using mathematical correction, this improved to 15.4% (0.02±0.03), with the proportion of misclassified stenoses falling to 6.7%. | 3 |
| 23 | Yang Y, Liu X, Xia Y, et al. Impact of spatial characteristics in the left stenotic coronary artery on the hemodynamics and visualization of 3D replica models. Sci Rep. 2017;7(1):15452. | Review/Other-Dx | 8 | In the present study, we performed anatomic and hemodynamic analysis on 8 left coronary arterial trees with 10 identified stenoses. | Our results suggested that the multiple spatial characteristics (curvature of the culprit vessel multiplied by an angle of the culprit's vessel to the upstream parent branch) could be an index of hemodynamics significance (r = -0.673, P-value = 0.033). and reduction of the maximum velocity from stenosis to downstream was found correlated to the FFRCT (r = 0.480, p = 0.160). In addition, 3D printed models could provide accurate replicas of the patient-specific left coronary arterial trees compare to virtual 3D models (r = 0.987, P-value < 0.001). | 4 |
| 24 | Sommer K, Izzo RL, Shepard L, et al. Design Optimization for Accurate Flow Simulations in 3D Printed Vascular Phantoms Derived from Computed Tomography Angiography. Proc SPIE Int Soc Opt Eng. 2017;10138. | Review/Other-Dx and Tx | 3 | We investigated methods to manage the distal arterial flow resistance and pressure thus creating physiologically and geometrically accurate phantoms that can be used for simulations of image-guided interventional procedures with new devices. | For the coronary phantom we obtained physiologically relevant waves which oscillated between 80 and 120 mmHg and a flow rate of ~125 ml/min, within the literature reported values. The pressure wave was similar with those acquired in human patients. | 4 |
| 25 | Shepard L, Sommer K, Izzo R, et al. Initial Simulated FFR Investigation Using Flow Measurements in Patient-specific 3D Printed Coronary Phantoms. Proc SPIE Int Soc Opt Eng. 2017;10138. | Review/Other-Dx | 4 | Accurate patient-specific phantoms for device testing or endovascular treatment planning can be 3D printed. We expand the applicability of this approach for cardiovascular disease, in particular, for CT-geometry derived benchtop measurements of Fractional Flow Reserve, the reference standard for determination of significant individual coronary artery atherosclerotic lesions. | 3D model based FFR measurements correlated well with stenosis severity. FFR measurements for each stenosis grade were: 0.8 severe, 0.7 moderate and 0.88 mild. | 4 |
| 26 | Shepard LM, Sommer KN, Angel E, et al. Initial evaluation of three-dimensionally printed patient-specific coronary phantoms for CT-FFR software validation. J Med Imaging (Bellingham). 2019;6(2):021603. | Observational-Dx | 5 | We developed three-dimensionally (3D) printed patient-specific coronary phantoms that are capable of sustaining physiological flow and pressure conditions. We assessed the accuracy of these phantoms from coronary CT acquisition, benchtop experimentation, and CT-FFR software. | Phantom diameter measurements were within 1 mm on average compared to patient measurements. Patient and phantom CT-FFR results had an absolute mean difference of 4.34% and Pearson correlation of 0.95. | 3 |
| 27 | Sommer KN, Shepard L, Karkhanis NV, et al. 3D Printed Cardiovascular Patient Specific Phantoms Used for Clinical Validation of a CT-derived FFR Diagnostic Software. Proc SPIE Int Soc Opt Eng. 2018;10578. | Observational-Dx | 12 | To demonstrate the utility of this approach we compared bench-top results with non-invasive CT-derived FFR software based on a computational fluid dynamics algorithm and catheter based FFR measurements. | All 12 patients completed the clinical study without any complication, and the three FFR techniques (Angio-FFR, CT-FFR, and Benchtop FFR) are reported for one or two main coronary arteries. The Pearson correlation among Benchtop FFR/Angio-FFR, CT-FFR/ Benchtop FFR, and CT-FFR/ Angio-FFR are 0.871, 0.877, and 0.927 respectively. | 2 |
| 28 | Sun Z, Jansen S. Personalized 3D printed coronary models in coronary stenting. Quant Imaging Med Surg. 2019;9(8):1356-1367. | Review/Other-Dx | 3 | In this study, we presented our experience of placing coronary stents into personalized 3D printed coronary models with the aim of determining stent lumen visibility with images reconstructed with different postprocessing views and algorithms. | All of these stents were successfully placed into the right and left coronary arteries but 2 of them did not obtain wall apposition along the complete length. The stent lumen visibility ranged from 54 to 97%, depending on the stent location in the coronary arteries. The mean stent lumen diameters measured on 2D axial, thin and thick slab MIP images were found to be significantly smaller than the actual size (P<0.01). Thick slab MIP images resulted in measured stent lumen diameters smaller than those from thin slab MIP images, with significant differences noticed in most of the measurements (4 out of 6 stents) (P<0.05), and no significant differences in the remaining 2 stents (P=0.19–0.38). In contrast, 3D volume rendering images allowed for more accurate measurements with measured stent diameters close to the actual dimensions in most of these coronary stents, except for the stent placed at the right coronary artery in one of the models due to insufficient expansion of the stent. Images reconstructed with sharp kernel Bv59 significantly improved stent lumen visibility when compared to the smooth Bv36 kernel (P=0.01). 3D VIE was successfully generated in all of the datasets with clear visualization of intraluminal views of the stents in relation to the coronary wall. | 4 |
| 29 | Lee M, Moharem-Elgamal S, Beckingham R, et al. Evaluating 3D-printed models of coronary anomalies: a survey among clinicians and researchers at a university hospital in the UK. BMJ Open. 2019;9(3):e025227. | Review/Other-Dx | N/A | To evaluate the feasibility of three-dimensional (3D) printing models of coronary artery anomalies based on cardiac CT data and explore their potential for clinical applications. | All models were reconstructed and printed successfully, with accurate details showing coronary anatomy (eg, anomalous coronary artery, coronary roofing or coronary aneurysm in a patient with Kawasaki syndrome). All clinicians and researchers provided feedback, with both groups finding the models helpful in displaying coronary artery anatomy and abnormalities, and complementary to viewing 3D CT scans. The clinicians’ group, who had substantially more imaging expertise, provided more enthusiastic ratings in terms of models’ clarity, usefulness and future use on average. | 4 |
| 30 | Oliveira-Santos M, Oliveira Santos E, Marinho AV, et al. Patient-specific 3D printing simulation to guide complex coronary intervention. Rev Port Cardiol. 2018;37(6):541 e541-541 e544. | Review/Other-Tx | 1 | We report the first case of a fully simulated percutaneous coronary intervention in a three-dimensional patient-specific model to guide treatment. | Procedural steps were replicated in the real patient's treatment, with remarkable parallelism in angiographic outcome and luminal gain at intracoronary imaging. | 4 |
| 31 | Salavitabar A, Chelliah A, Kalfa D, Crystal MA. When a coronary artery fistula is not simply a fistula: Using multimodality imaging to demonstrate an unusual embryologic remnant. J Thorac Cardiovasc Surg. 2018;156(1):358-362. | Review/Other-Tx | 1 | Three-dimensional models were digitally created and printed with hollowed-out vessel lumens for testing of catheter courses and predetermined fluoroscopic angles | No abstract available. | 4 |
| 32 | Wang H, Liu J, Zheng X, et al. Three-dimensional virtual surgery models for percutaneous coronary intervention (PCI) optimization strategies. Sci Rep. 2015;5:10945. | Review/Other-Dx and Tx | N/A | To optimize the stent position for lowering the risk of restenosis, we successfully established a digital three-dimensional (3-D) model based on a real clinical coronary artery and analysed the optimal stenting strategies by computational simulation. | Simultaneously, physicians placed real stents inside them; i.e., they performed "virtual surgeries". The hydrodynamic experimental results showed that the microfluidic models highly inosculated the simulations. | 4 |
| 33 | Misra A, Walters HL, Kobayashi D. Utilisation of a three-dimensional printed model for the management of coronary-pulmonary artery fistula from left main coronary artery. Cardiol Young. 2019;29(3):431-434. | Review/Other-Tx | 1 | In the presented case, a coronary-pulmonary artery fistula arose from the left main coronary artery and supplied blood flow to a left upper lobe segment. | The life-sized three-dimensional printed model was helpful in pre-surgical planning for unifocalisation of the aortopulmonary collateral arteries. | 4 |
| 34 | Aroney N, Lau K, Daniele L, Burstow D, Walters D. Three-dimensional printing: to guide management of a right coronary artery to left ventricular fistula. Eur Heart J Cardiovasc Imaging. 2018;19(3):268. | Review/Other-Dx | 8 | To evaluate the feasibility of three-dimensional (3D) printing models of coronary artery anomalies based on cardiac CT data and explore their potential for clinical applications. | All models were reconstructed and printed successfully, with accurate details showing coronary anatomy (eg, anomalous coronary artery, coronary roofing or coronary aneurysm in a patient with Kawasaki syndrome). All clinicians and researchers provided feedback, with both groups finding the models helpful in displaying coronary artery anatomy and abnormalities, and complementary to viewing 3D CT scans. The clinicians’ group, who had substantially more imaging expertise, provided more enthusiastic ratings in terms of models’ clarity, usefulness and future use on average. | 4 |
| 35 | Velasco Forte MN, Byrne N, Valverde Perez I, et al. 3D printed models in patients with coronary artery fistulae: anatomical assessment and interventional planning. EuroIntervention. 2017;13(9):e1080-e1083. | Observational-Dx and Tx | 4 | Coronary artery fistulae represent one of the most challenging anatomical defects to define accurately. We aimed to investigate the additional benefit conferred by volume rendering of tomographic images and 3D printing for diagnosis and interventional planning. | All diagnoses and planned management were reviewed after inspection of a 3D model. Using source images alone, both cardiologists correctly described the course and drainage in two out of four cases. Aided by volume rendering, this improved to three out of four cases. Inspection of the 3D printed model prompted the planned interventional approach and device sizing to be altered in two out of four cases. In one out of four cases, the intervention was abandoned after inspection of the 3D printed model. | 3 |
| 36 | Sedaghat A, Wolpers AC, Menne M, et al. Percutaneous treatment of a saccular coronary artery aneurysm using multimodal imaging and rapid prototyping. Eur Heart J. 2018;39(46):4125. | Review/Other-1Tx | 1 | Additionally rapid prototyping was used to produce a silicone model to allow ex vivo evaluation and visualization | No abstract available. | 4 |
| 37 | Lazkani M, Bashir F, Brady K, Pophal S, Morris M, Pershad A. Postinfarct VSD management using 3D computer printing assisted percutaneous closure. Indian Heart J. 2015;67(6):581-585. | Review/Other-Tx | 1 | Although percutaneous PIVSD closure has been reported in the literature, this is the first case report where 3D printing and modeling were used to guide closure. | No results stated in abstract. | 4 |
| 38 | Mohamed E, Telila T, Osaki S, Jacobson K. Percutaneous closure of left ventricle pseudoaneurysm using 3D printed heart model for procedure planning: A novel approach. Catheter Cardiovasc Interv. 2019. | Review/Other-Tx | 1 | In this report, we present a case of successful percutaneous closure of left ventricle pseudoaneurysm using 3D printing for procedure guidance. | No results stated in abstract. | 4 |
| 39 | So CY, Fan Y, Wu EB, Lee AP. 3D Printing in Transcatheter Aortic Valve Implantation: Anticipating Coronary Obstruction in High Risk Aortic Root Anatomy. EuroIntervention. 2019. | Review/Other-Tx | 3 | Simulation of transcatheter aortic valve (AV) implantation (TAVI) on three-dimensional (3D) printed models were performed in 3 patients with high risk anatomy for coronary obstruction (CO). | No abstract available. | 4 |
| 40 | Bompotis G, Meletidou M, Karakanas A, et al. Transcatheter Aortic Valve Implantation using 3-D printing modeling assistance. A single-center experience. Hellenic J Cardiol. 2019. | Review/Other-Dx and Tx | 1 | An innovative imaging modality – the 3D printing modeling – was used for the aortic valve and the contiguous parts of the heart, aorta, and coronary arteries. | No abstract available. | 4 |
| 41 | Yamawaki M, Obama K, Sasuga S, et al. Underfilled Balloon-Expandable Transcatheter Aortic Valve Implantation With Ad Hoc Post-Dilation- Pulsatile Flow Simulation Using a Patient-Specific Three-Dimensional Printing Model. Circ J. 2019;83(2):461-470. | Review/Other-Tx | 1 | Underfilled transcatheter aortic-valve implantation with ad hoc post-dilation is a therapeutic option for patients with borderline annuli to avoid acute complication. The effects of this technique on valve leaflet behavior, hydrodynamic performances, and paravalvular leakage (PVL) using patient-specific three-dimensional (3D) aortic-valve models were investigated. | Observation using a high-speed camera revealed distorted leaflets after underfilled implantation, with a longer valve-closing time and smaller effective orifice areas, especially in the -3 cc underfilled implantation. Micro-CT analysis revealed that the transcatheter valves shifted to the opposite side of the large annulus calcification after post-dilation and reduced the malapposition there. | 4 |
| 42 | Gomes EN, Dias RR, Rocha BA, et al. Use of 3D Printing in Preoperative Planning and Training for Aortic Endovascular Repair and Aortic Valve Disease. Braz J Cardiovasc Surg. 2018;33(5):490-495. | Review/Other-Tx | 6 | Six 3D printed real scale prototypes were built representing different aortic diseases, taken from real patients, to simulate the correction of the disease with endoprosthesis deployment. | In the hybrid room, the 3D prototypes were examined under fluoroscopy, making it possible to obtain images that clearly delimited the walls of the aorta and its details. The endovascular simulation was then able to be performed, by correctly positioning the endoprosthesis, followed by its deployment. | 4 |
| 43 | Hosny A, Dilley JD, Kelil T, et al. Pre-procedural fit-testing of TAVR valves using parametric modeling and 3D printing. J Cardiovasc Comput Tomogr. 2019;13(1):21-30. | Review/Other-Tx | 30 | To improve this understanding, we developed a benchtop workflow that allows for testing of physical interactions between prosthetic valves and patient-specific aortic root anatomy, including calcified leaflets, prior to actual prosthetic valve placement. | Benchtop-predicted "best fit" valve size showed a statistically significant correlation with gold standard CT measurements of the average annulus diameter (n = 30, p < 0.0001 Wilcoxon matched-pairs signed rank test). Adequateness of seal (presence or absence of paravalvular leak) was correctly predicted in 11/15 (73.3%) patients who received a balloon-expandable valve, and in 9/15 (60%) patients who received a self-expanding valve. Pressure testing provided a physical map of areas with an inadequate seal; these corresponded to areas of paravalvular leak documented by post-procedural transthoracic echocardiography. | 4 |
| 44 | Hatoum H, Dollery J, Lilly SM, Crestanello JA, Dasi LP. Sinus Hemodynamics Variation with Tilted Transcatheter Aortic Valve Deployments. Ann Biomed Eng. 2019;47(1):75-84. | Review/Other-Tx | 1 | This study aims to elucidate TAV angular misalignment with respect to aortic root axis effect on sinus flow stasis potentially leading to leaflet thrombosis. | While pressure gradients differed insignificantly, blood velocity and vorticity decreased significantly in both tilted cases sinuses. Shear stress probability near the leaflet decreases with tilt indicating stasis. TAV tilted away from the sinus is the most unfavorable scenario with poor washout. | 4 |
| 45 | Harb SC, Xu B, Klatte R, Griffin BP, Rodriguez LL. Haemodynamic Assessment of Severe Aortic Stenosis Using a Three-Dimensional (3D) Printed Model Incorporating a Flow Circuit. Heart Lung Circ. 2018;27(11):e105-e107. | Review/Other-Tx | 1 | Here, we demonstrate a novel application of 3D printing in the haemodynamic assessment of severe aortic stenosis, using a flow circuit. | No abstract available. | 4 |
| 46 | Faletti R, Gatti M, Cosentino A, et al. 3D printing of the aortic annulus based on cardiovascular computed tomography: Preliminary experience in pre-procedural planning for aortic valve sizing. J Cardiovasc Comput Tomogr. 2018;12(5):391-397. | Review/Other-Tx | 20 | To determine reliability and reproducibility of measurements of aortic annulus in 3D models printed from cardiovascular computed tomography (CCT) images. | No significant differences were found among the measurements made by each cardiac surgeon on the same 3D model (p = 0.48) or on the 3D models printed by different manufacturers (p = 0.25); also, no intraobserver variability (p = 0.46). The annulus diameter measured on 3D models showed good agreement with the reference CCT measurement (p = 0.68) and IOH sizing (p = 0.11). Time and cost per model were: model creation ∼10-15 min; printing time ∼60 min; post-processing ∼5min; material cost ∼1€. | 4 |
| 47 | Shirakawa T, Yoshitatsu M, Koyama Y, Mizoguchi H, Toda K, Sawa Y. 3D-printed aortic stenosis model with fragile and crushable calcifications for off-the-job training and surgical simulation. Multimed Man Cardiothorac Surg. 2018;2018. | Review/Other-Tx | 1 | In this tutorial we present our method for creating an aortic stenosis model with realistically fragile and crushable calcifications, using modern 3D-printing techniques. | No results stated in abstract. | 4 |
| 48 | Zhou X, Vannan MA, Qian Z. 3D printing for trans-catheter aortic valve replacement: Integrating anatomy and physiology to plan, predict and optimize procedural outcomes. Int J Cardiol. 2018;258:334-335. | Review/Other-Tx | NA | Comment and suggestion on another study | No abstract available. | 4 |
| 49 | Alkhouli M, Sengupta PP. 3-Dimensional-Printed Models for TAVR Planning: Why Guess When You Can See? JACC Cardiovasc Imaging. 2017;10(7):732-734. | Review/Other-Tx | NA | Comment and suggestion on another study | No abstract available. | 4 |
| 50 | Hernandez-Enriquez M, Brugaletta S, Andreu D, et al. Three-dimensional printing of an aortic model for transcatheter aortic valve implantation: possible clinical applications. Int J Cardiovasc Imaging. 2017;33(2):283-285. | Review/Other-Tx | 1 | Finally, a Witbox-2 3D printer (bq, Madrid, Spain) was used to print both the prothesis library and the 3D model of the aortic root. | No abstract available. | 4 |
| 51 | Fujita B, Kutting M, Scholtz S, et al. Development of an algorithm to plan and simulate a new interventional procedure. Interact Cardiovasc Thorac Surg. 2015;21(1):87-95. | Review/Other-Tx | 1 | The aim of this study was to develop a feasible algorithm to plan and in vitro simulate a new interventional procedure to improve patient outcome. | This algorithm was developed for a patient with a degenerated Perceval aortic sutureless prosthesis requiring a ViV procedure. Different ViV procedures were assessed in the algorithm and based on these results the best option for the patient was chosen. The actual procedure went exactly as planned with help of this algorithm. | 4 |
| 52 | Fujita B, Kutting M, Seiffert M, et al. Calcium distribution patterns of the aortic valve as a risk factor for the need of permanent pacemaker implantation after transcatheter aortic valve implantation. Eur Heart J Cardiovasc Imaging. 2016;17(12):1385-1393. | Review/Other-Tx | 2 | The aim of this study was to identify calcification patterns with an elevated risk for permanent pacemaker implantation (PPI) after TAVI and investigate underlying mechanisms in an ex vivo setting. | Patients with a calcium load of the left coronary cusp (LCC) above 209 mm3 had a higher rate of PPI than patients below this threshold (16.7 vs. 2.6%, P = 0.003). Multivariate regression revealed pre-existing right bundle branch block (RBBB) and increased LCC calcification as independent predictors for PPI. Simulation of the TAVI procedure in a silicone annulus revealed an off-centreline shift of the valvuloplasty balloon and transcatheter heart valve away from the LCC towards the commissure between right- and non-coronary cusp. | 4 |
| 53 | Fujita T, Saito N, Minakata K, Imai M, Yamazaki K, Kimura T. Transfemoral transcatheter aortic valve implantation in the presence of a mechanical mitral valve prosthesis using a dedicated TAVI guidewire: utility of a patient-specific three-dimensional heart model. Cardiovasc Interv Ther. 2017;32(3):308-311. | Review/Other-Tx | 1 | Thus, we conducted a simulation using a three-dimensional heart model to confirm the safety of the procedure. | The procedure was successful without any complications. | 4 |
| 54 | Jung JI, Koh YS, Chang K. 3D Printing Model before and after Transcatheter Aortic Valve Implantation for a Better Understanding of the Anatomy of Aortic Root. Korean Circ J. 2016;46(4):588-589. | Review/Other-Tx | 1 | Using the CT data, a 3D printing model was created (Materialise®, Luven, Belgium). 3D printing model provided the cardiologist with an intuitive perception of the cardiovascular anatomy and measurements in preparation for TAVI | No abstract available. | 4 |
| 55 | Gallo M, D'Onofrio A, Tarantini G, Nocerino E, Remondino F, Gerosa G. 3D-printing model for complex aortic transcatheter valve treatment. Int J Cardiol. 2016;210:139-140. | Review/Other-Tx | 1 | 3D-printed cardiovascular models can be particularly helpful to “the heart team” by providing a custom-made representation of the heart before an interventional procedure. | No abstract available. | 4 |
| 56 | Ripley B, Kelil T, Cheezum MK, et al. 3D printing based on cardiac CT assists anatomic visualization prior to transcatheter aortic valve replacement. J Cardiovasc Comput Tomogr. 2016;10(1):28-36. | Review/Other-Dx and Tx | 16 | To determine the feasibility of using cardiac CT to print individual models of the aortic root complex for transcatheter aortic valve replacement (TAVR) planning as well as to determine the ability to predict paravalvular aortic regurgitation (PAR). | Aortic root 3D models were highly accurate, with excellent agreement between annulus measurements made on 3D models and those made on corresponding 2D data (mean difference of -0.34 mm, 95% limits of agreement: ± 1.3 mm). The 3D printed valve models were within 0.1 mm of their designed dimensions. Examination of the fit of valves within patient-specific aortic root models correctly predicted PAR in 6 of 9 patients (6 true positive, 3 false negative) and absence of PAR in 5 of 7 patients (5 true negative, 2 false positive). | 4 |
| 57 | Maragiannis D, Jackson MS, Igo SR, et al. Replicating Patient-Specific Severe Aortic Valve Stenosis With Functional 3D Modeling. Circ Cardiovasc Imaging. 2015;8(10):e003626. | Review/Other-Tx | 8 | We sought to apply 3D printing technologies to develop patient-specific models of the anatomic and functional characteristics of severe aortic valve stenosis. | Doppler-derived measures of peak and mean transvalvular gradient correlated well with reference standard pressure catheters across a range of flow conditions (r=0.988 and r=0.978 respectively, P<0.001). Aortic valve orifice area by Gorlin and Doppler methods correlated well (r=0.985, P<0.001). Calculated aortic valve area increased a small amount for both methods with increasing flow (P=0.002). | 4 |
| 58 | Schmauss D, Schmitz C, Bigdeli AK, et al. Three-dimensional printing of models for preoperative planning and simulation of transcatheter valve replacement. Ann Thorac Surg. 2012;93(2):e31-33. | Review/Other-Tx | 1 | In this study, we show the use of three-dimensional printing models for preoperative planning of transcatheter valve replacement in a patient with an extreme porcelain aorta. | Therefore, we decided to fabricate three-dimensional models to evaluate the potential effects of these constructs for previous surgical planning and simulation of the transcatheter valve replacement. | 4 |
| 59 | Rotman OM, Kovarovic B, Sadasivan C, Gruberg L, Lieber BB, Bluestein D. Realistic Vascular Replicator for TAVR Procedures. Cardiovasc Eng Technol. 2018;9(3):339-350. | Review/Other-Tx | NA | We report on a novel benchtop patient-specific arterial replicator designed for testing TAVR and training interventional cardiologists in the procedure. | In the Replicator of both valves were compared to the performance in a commercial ISO-compliant LHS. The AS anatomy in the Replicator resulted in a significant decrease of the TAVR valve performance relative to the simplified LHS, with EOA and transvalvular pressures comparable to clinical data. Minor change was seen in the mechanical valve performance. | 4 |
| 60 | Benke K, Barabas JI, Daroczi L, et al. Routine aortic valve replacement followed by a myriad of complications: role of 3D printing in a difficult cardiac surgical case. J Thorac Dis. 2017;9(11):E1021-E1024. | Review/Other-Tx | 1 | In this report, we present a complex and unique case following AVR in a middle-aged woman. | No results stated in abstract. | 4 |
| 61 | Jelenc M, Jelenc B, Knezevic I, Klokocovnik T. New graft sizing rings for aortic valve reimplantation procedures. Interact Cardiovasc Thorac Surg. 2018;26(1):1-3. | Observational-Tx | 10 | The objective was to design sizing rings that would enable proper sizing of the graft in reimplantation procedures and to perform leaflet repair before graft implantation. | The rings were successfully used in 10 of our latest reimplantation procedures. After dissection of the aortic root, the commissures were suspended with U-stitches and then the ring was seated onto them. Complete leaflet repair with plication to achieve adequate effective height was then performed, followed by graft implantation. No additional leaflet repair was needed. | 3 |
| 62 | Sodian R, Schmauss D, Markert M, et al. Three-dimensional printing creates models for surgical planning of aortic valve replacement after previous coronary bypass grafting. Ann Thorac Surg. 2008;85(6):2105-2108. | Review/Other-Tx | 1 | Thus, we are exploring the effect of using rapid prototyping techniques for surgical planning and intraoperative orientation during aortic valve replacement after previous coronary artery bypass grafting (CABG). | Using data derived from 128-slice computed tomography angiography linked to proprietary software, we were able to create three-dimensional reconstructions of the vascular anatomy after the previous CABG. The models were sterilized and taken to the operating theatre for orientation during the surgical procedure. | 4 |
| 63 | Baribeau Y, Sharkey A, Mahmood E, et al. Three-Dimensional Printing and Transesophageal Echocardiographic Imaging of Patient-Specific Mitral Valve Models in a Pulsatile Phantom Model. J Cardiothorac Vasc Anesth. 2019. | Review/Other-Tx | NA | Herein a customized pulsatile left-sided heart model that uses patient-specific 3-dimensional printed valves under physiological intracardiac pressures as a TEE task trainer is described. | No results stated in abstract. | 4 |
| 64 | Ginty OK, Moore JT, Eskandari M, et al. Dynamic, patient-specific mitral valve modelling for planning transcatheter repairs. Int J Comput Assist Radiol Surg. 2019;14(7):1227-1235. | Review/Other-Dx and Tx | 6 | We aim to produce a workflow for manufacturing dynamic patient-specific models to simulate the mitral valve for transcatheter repair applications. | Early results indicate the workflow has excellent anatomical accuracy and the ability to replicate regurgitation pathologies, as shown by colour Doppler ultrasound and anatomical measurements comparing patients and models. Analysis of all measurements successfully resulted in t critical two-tail > t stat and p values > 0.05, thus demonstrating no statistical difference between the patients and models, owing to high fidelity morphological replication. | 4 |
| 65 | Kohli K, Wei ZA, Yoganathan AP, Oshinski JN, Leipsic J, Blanke P. Transcatheter Mitral Valve Planning and the Neo-LVOT: Utilization of Virtual Simulation Models and 3D Printing. Curr Treat Options Cardiovasc Med. 2018;20(12):99. | Review/Other-Tx | NA | The goal of this review is to provide an overview of key concepts relating to TMVR pre-procedural planning, with particular emphasis on imaging-based methods for predicting TMVR-related LVOT obstruction. | No results stated in abstract. | 4 |
| 66 | Ferrari E, Biasco L, Faletra F, et al. Tiara Valve Implantation in a Patient With Previously Implanted Mono-disk Mechanical Aortic Prosthesis. Semin Thorac Cardiovasc Surg. 2018;30(2):160-163. | Review/Other-Tx | 1 | Hereafter, we describe the case of a patient with a 27-mm mono-disk mechanical aortic valve implanted in 1978, a EuroSCORE II of 18%, and a Society of Thoracic Surgeon score (mortality) of 16% who successfully underwent a transapical Tiara valve implantation. | No results stated in abstract. | 4 |
| 67 | Bagur R, Cheung A, Chu MWA, Kiaii B. 3-Dimensional-Printed Model for Planning Transcatheter Mitral Valve Replacement. JACC Cardiovasc Interv. 2018;11(8):812-813. | Review/Other-Tx | 1 | Hence, we simulated TMV-in-R replacement by positioning a TIARA valve within the mitral ring of the patient-specific 3D-printed phantom model to predict adequate clearance between the aortic leaflets and the proposed TIARA-TMV | No abstract available. | 4 |
| 68 | El Sabbagh A, Eleid MF, Matsumoto JM, et al. Three-dimensional prototyping for procedural simulation of transcatheter mitral valve replacement in patients with mitral annular calcification. Catheter Cardiovasc Interv. 2018;92(7):E537-E549. | Review/Other-Tx | 5 | Three-dimensional (3D) prototyping is a novel technology which can be used to plan and guide complex procedures such as transcatheter mitral valve replacement (TMVR). | 3D prototyping simulated LVOT obstruction in one patient who developed it and in another patient who underwent alcohol septal ablation prior to TMVR. Valve sizing correlated with actual placed valve size in six out of the eight patients and more than mild paravalvular leak (PVL) was simulated in two of the three patients who had it. Patients who had mismatch between their modeled valve size and post-procedural imaging were the ones that had anterior leaflet resection which could have altered valve sizing and PVL simulation. 3D printed model of one of the latter patients allowed modification of anterior leaflet to simulate surgical resection and was able to estimate the size and location of the PVL after inserting a valve stent into the physical model. | 4 |
| 69 | Wang DD, Eng MH, Greenbaum AB, et al. Validating a prediction modeling tool for left ventricular outflow tract (LVOT) obstruction after transcatheter mitral valve replacement (TMVR). Catheter Cardiovasc Interv. 2018;92(2):379-387. | Observational-Tx | 38 | Demonstrate proof-of-concept validation of a computed tomography (CT) computer-aided design prediction modeling tool to identify patients at risk for left ventricular outflow tract (LVOT) obstruction in transcatheter mitral valve replacement (TMVR). | All patients underwent successful TMVR without device embolization. Seven of the 38 patients experienced LVOT obstruction, defined as an increase of ≥10 mmHg LVOT peak gradient post-TMVR. Anatomic screening using CT was validated in 20/38 patients as preprocedural predicted neo-LVOT surface area correlated well with post-TMVR measurements (R2  = 0.8169, P < 0.0001). A receiver operating curve curve found a predicted neo-LVOT surface area of ≤ 189.4 mm2 to have 100% sensitivity and 96.8% specificity for predicting TMVR-induced LVOT obstruction. | 3 |
| 70 | Ginty O, Moore J, Peters T, Bainbridge D. Modeling Patient-Specific Deformable Mitral Valves. J Cardiothorac Vasc Anesth. 2018;32(3):1368-1373. | Review/Other-Tx | NA | The authors describe the generalized process of creating a model of cardiac anatomy from patient images and their experience creating patient-specific dynamic mitral valve models. | No results stated in abstract. | 4 |
| 71 | Scanlan AB, Nguyen AV, Ilina A, et al. Comparison of 3D Echocardiogram-Derived 3D Printed Valve Models to Molded Models for Simulated Repair of Pediatric Atrioventricular Valves. Pediatr Cardiol. 2018;39(3):538-547. | Review/Other-Tx | 5 | We compared materials, processes, and costs for 3D printing and molding of patient-specific models for visualization and surgical simulation of congenitally abnormal heart valves. | Surgeon assessment indicated that the molded valves had superior material properties for the purposes of simulation compared to directly printed valves (p < 0.01). | 4 |
| 72 | Zhu Y, Liu J, Wang L, et al. Preliminary study of the application of transthoracic echocardiography-guided three-dimensional printing for the assessment of structural heart disease. Echocardiography. 2017;34(12):1903-1908. | Observational-Dx and Tx | 44 | To investigate the feasibility and diagnostic value of a preoperative transthoracic echocardiography-guided three-dimensional printed model (TTE-guided 3DPM) for the assessment of structural heart disease (SHD). | The sensitivity and specificity of the TTE-guided 3DPM were greater than or equal to those of the 3DTTE. The P-value of the McNemar test of 3DTTE was >.05, which indicates that the difference was not statistically significant (Kappa = 0.745, P < .001). The P-value of the McNemar test of TTE-guided 3DPM was >.05, which indicates that the difference was not statistically significant (Kappa = 0.955, P < .001). A comparison of 3DTTE and TTE-guided 3DPM resulted in a P-value >.05, which indicates that the difference was not statistically significant (Kappa = 0.879, P < .001). TTE-guided 3DPM displayed the 3D structure of SHDs and cardiac lesions clearly and was consistent with the intra-operative findings. | 2 |
| 73 | Dahle G, Rein KA, Fiane AE. Single centre experience with transapical transcatheter mitral valve implantation. Interact Cardiovasc Thorac Surg. 2017;25(2):177-184. | Observational-Tx | 11 | We present our single centre experience with transcatheter mitral valve implantation-transapical approach procedures in eleven patients. | Implantation success was 100% with no left ventricular outflow tract obstruction. Good haemodynamics and improved New York Heart Association class were demonstrated in all patients. One patient died before 30 days due to sepsis. One patient had a valve thrombosis when switching from Coumadin to new oral anticoagulant and had a second valve implanted into the first one as a 'valve-in-valve' procedure. | 2 |
| 74 | Vukicevic M, Puperi DS, Jane Grande-Allen K, Little SH. 3D Printed Modeling of the Mitral Valve for Catheter-Based Structural Interventions. Ann Biomed Eng. 2017;45(2):508-519. | Observational-Tx | 3 | Our aim with this investigation was to combine the technologies of high-spatial resolution cardiac imaging, image processing software, and fused multi-material 3D printing, to demonstrate that patient-specific models of the mitral valve apparatus could be created to facilitate functional evaluation of novel trans-catheter mitral valve repair strategies. | The effective bending modulus of healthy porcine MV tissue was significantly less than the bending modulus of TangoPlus (p < 0.01). All TangoPlus varieties were less stiff than the maximum tensile elastic modulus of mitral valve tissue (3697.2 ± 385.8 kPa anterior leaflet; 2582.1 ± 374.2 kPa posterior leaflet) (p < 0.01). However, the slopes of the stress-strain toe regions of the mitral valve tissues (532.8 ± 281.9 kPa anterior leaflet; 389.0 ± 156.9 kPa posterior leaflet) were not different than those of the Shore 27, Shore 35, and Shore 27 with Shore 35 blend TangoPlus material (p > 0.95). | 3 |
| 75 | Mashari A, Knio Z, Jeganathan J, et al. Hemodynamic Testing of Patient-Specific Mitral Valves Using a Pulse Duplicator: A Clinical Application of Three-Dimensional Printing. J Cardiothorac Vasc Anesth. 2016;30(5):1278-1285. | Review/Other-Tx | Not clearly stated | To evaluate the feasibility of obtaining hemodynamic metrics of echocardiographically derived 3-dimensional printed mitral valve models deployed in a pulse-duplicator chamber. | The authors were able to obtain continuous-wave Doppler tracings of the valve inflow with a transesophageal echocardiography transducer. They also were able to generate diastolic ventricular and atrial pressure tracings. Pressure half-time and mitral valve area were computed from these measurements. | 4 |
| 76 | Little SH, Vukicevic M, Avenatti E, Ramchandani M, Barker CM. 3D Printed Modeling for Patient-Specific Mitral Valve Intervention: Repair With a Clip and a Plug. JACC Cardiovasc Interv. 2016;9(9):973-975. | Review/Other-Tx | 1 | A 3-dimensional (3D) printed multimaterial model of the mitral valve leaflets and subvalvular calcium deposition was created to facilitate selection and sizing of an occluder device for the posterior leaflet perforation. | No abstract available. | 4 |
| 77 | Izzo RL, O'Hara RP, Iyer V, et al. 3D Printed Cardiac Phantom for Procedural Planning of a Transcatheter Native Mitral Valve Replacement. Proc SPIE Int Soc Opt Eng. 2016;9789. | Review/Other-Tx | 1 | 3D printing an anatomically accurate, functional flow loop phantom of a patient's cardiac vasculature was used to assist in the surgical planning of one of the first native transcatheter mitral valve replacement (TMVR) procedures. | After performing the mock-procedure on the cardiac phantom, the cardiologists optimized their transapical surgical approach. The mitral valve stenosis and calcification were clearly visible. The phantom was used to inform the sizing of the valve to be implanted. | 4 |
| 78 | Mahmood F, Owais K, Taylor C, et al. Three-dimensional printing of mitral valve using echocardiographic data. JACC Cardiovasc Imaging. 2015;8(2):227-229. | Review/Other-Tx | Not clearly stated | Using 3D electrocardiogram-gated volumetric TEE datasets, we assessed the feasibility of printing models of normal, ischemic, and myxomatous MVs. | No abstract available. | 4 |
| 79 | Owais K, Pal A, Matyal R, et al. Three-dimensional printing of the mitral annulus using echocardiographic data: science fiction or in the operating room next door? J Cardiothorac Vasc Anesth. 2014;28(5):1393-1396. | Review/Other-Tx | Not clearly stated | In this paper, the authors present a novel method of creating patient-specific 3D printed solid models of the mitral valve annulus using 3D TEE data. | No abstract available. | 4 |
| 80 | Witschey WR, Pouch AM, McGarvey JR, et al. Three-dimensional ultrasound-derived physical mitral valve modeling. Ann Thorac Surg. 2014;98(2):691-694. | Review/Other-Tx | 4 | To educate and guide repair surgery further, we have developed a methodology for fast production of physical models of the valve using novel three-dimensional (3D) echocardiographic imaging software in combination with stereolithographic printing. | Physical models of normal and diseased valves (ischemic mitral regurgitation and myxomatous degeneration) were constructed. There was good correspondence between the virtual shape models and physical model | 4 |
| 81 | Hascoet S, Smolka G, Bagate F, et al. Multimodality imaging guidance for percutaneous paravalvular leak closure: Insights from the multi-centre FFPP register. Arch Cardiovasc Dis. 2018;111(6-7):421-431. | Review/Other-Tx | 2 | To describe imaging modalities used in clinical practice to guide percutaneous PVL closure and assess the potential of new imaging tools. | Data from 173 procedures performed in 19 centres from three countries (France, Belgium and Poland) were collected, which included eight cases of PVL following transcatheter valve replacement. Transoesophageal echocardiography was used in 167 cases (96.5%) and 3D echocardiography in 87.4% of cases. In one case, 3D-echocardiography was fused with fluoroscopy images in real time using echonavigator software. Details about multimodality imaging were available from a sample of 31 patients. Cardiac computed tomography (CT) was performed before 10 of the procedures. In one case, fusion between preprocedural cardiac CT angiography data and fluoroscopy data was used. In two cases, a 3D model of the valve with PVL was printed. | 4 |
| 82 | Engelhardt S, Sauerzapf S, Preim B, Karck M, Wolf I, De Simone R. Flexible and comprehensive patient-specific mitral valve silicone models with chordae tendineae made from 3D-printable molds. Int J Comput Assist Radiol Surg. 2019;14(7):1177-1186. | Review/Other-Tx | 9 | In order to provide a realistic tool for surgical training, a commercial simulator was augmented by flexible patient-specific mitral valve replica. | To our knowledge, our models are the first to comprise the full mitral valve apparatus, i.e., the annulus, leaflets, chordae tendineae and papillary muscles. Nine different valve molds were automatically created according to the proposed workflow (seven prolapsed valves and two valves with functional mitral insufficiency). From these mold geometries, 16 replica were manufactured. A material test revealed that EcoflexTM 00-30 is the most suitable material for leaflet-mimicking tissue out of seven mixtures. Production time was around 36 h per valve. Twelve surgeons performed various surgical techniques, e.g., annuloplasty, neo-chordae implantation, triangular leaflet resection, and assessed the realism of the valves very positively. | 4 |
| 83 | Theodoropoulos KC, Papachristidis A, Fonseca T, Reiken J, Monaghan MJ. Understanding the anatomy of a perforated mitral valve: From 2D echocardiography to 3D printing. Hellenic J Cardiol. 2018. | Review/Other-Tx | 1 | A 3D model of the mitral valve was created with 3D printing, providing anatomical details of the lesion. | No results stated in abstract. | 4 |
| 84 | Daemen JHT, Heuts S, Olsthoorn JR, Maessen JG, Sardari Nia P. Mitral valve modelling and three-dimensional printing for planning and simulation of mitral valve repair. Eur J Cardiothorac Surg. 2019;55(3):543-551. | Observational-Tx | 10 | The aim of this study was to develop a process for modelling and 3-dimensional (3D) printing of different mitral valve diseases for procedural planning and simulation, based on 3D transoesophageal echocardiography (TOE). | The mitral valves of 10 prospective patients with different diseases were modelled. In 6 patients, a 3D printed rigid plastic mitral valve was created for procedural planning, and in 4 patients, a silicone-cast replica was created for procedural simulation. All models were created to scale, implying conservation of in vivo dimensions. Models were validated by in vivo comparison. Total workaround time ranged from 3 to 4 h and 2 to 3 days for rigid plastic and silicone models, respectively. Costs were €15 to €40 and €300, respectively. | 3 |
| 85 | Ginty OK, Moore JM, Xu Y, et al. Dynamic Patient-Specific Three-Dimensional Simulation of Mitral Repair: Can We Practice Mitral Repair Preoperatively? Innovations (Phila). 2018;13(1):11-22. | Review/Other-Tx | 10 | We propose that three-dimensional printed, patient-specific, dynamic mitral valve models may help surgeons plan and trial all the details of a specific patient's mitral repair preoperatively. | Model measurements were accurate when compared with patients on anterior-posterior diameter, circumference, and anterior leaflet length; however, less accurate on posterior leaflet length. On subjective assessment, Likert scores were high at 3.8 ± 0.4 and 3.4 ± 0.7, suggesting good fidelity of the dynamic model echocardiogram and functional model in the phantom to the preoperative three-dimensional echocardiogram, respectively. Mitral repair was successful in all 10 models with significant reduction in mitral insufficiency. In two models, mitral repair was performed twice, using two different surgical techniques to assess which provided a better outcome. When compared with the actual patient mitral repair outcome, the repaired models compared favorably. | 4 |
| 86 | Yamada T, Osako M, Uchimuro T, et al. Three-Dimensional Printing of Life-Like Models for Simulation and Training of Minimally Invasive Cardiac Surgery. Innovations (Phila). 2017;12(6):459-465. | Review/Other-Tx | NA | We developed a heart model for simulation and training of minimally invasive cardiac surgery, particularly minimally invasive mitral valve repair using our new three-dimensional printing system. | We investigated the elastic modulus and breaking strength of the porcine heart. Based on investigation results, the cardiac model was set at rupture strength 20 MPa, elastic modulus 0.17 MPa, and moisture content 85%. This provided a biotexture and feeling exactly like a patient heart. Computed tomography scans confirmed that the model shape was nearly the same as that of a human heart. We simulated minimally invasive mitral valve repair, including ring annuloplasty, chordal reconstruction, resection and suture, and edge-to-edge repair. Full surgery simulations using this model used minimally invasive cardiac surgery tools including a robot. | 4 |
| 87 | Premyodhin N, Mandair D, Ferng AS, et al. 3D printed mitral valve models: affordable simulation for robotic mitral valve repair. Interact Cardiovasc Thorac Surg. 2018;26(1):71-76. | Review/Other-Tx | NA | 3D printed mitral valve (MV) models that capture the suture response of real tissue may be utilized as surgical training tools. Leveraging clinical imaging modalities, 3D computerized modelling and 3D printing technology to produce affordable models complements currently available virtual simulators and paves the way for patient- and pathology-specific preoperative rehearsal. | Valves produced with the moulding and casting method maintained anatomical dimensions within 3% of directly 3D printed acrylonitrile butadiene styrene controls for both morphologies. Likert-type scale mean scores corresponded with a realistic material response to sutures (5.0/5), tensile strength that is similar to real MV tissue (5.0/5) and anatomical appearance resembling real MVs (5.0/5), indicating that evaluators 'agreed' that these aspects of the model were appropriate for training. Evaluators 'somewhat agreed' that the overall model durability was appropriate for training (4.0/5) due to the mounting design. Qualitative differences in repair quality were notable between fellow and attending surgeon. | 4 |
| 88 | Sardari Nia P, Heuts S, Daemen J, et al. Preoperative planning with three-dimensional reconstruction of patient's anatomy, rapid prototyping and simulation for endoscopic mitral valve repair. Interact Cardiovasc Thorac Surg. 2017;24(2):163-168. | Review/Other-Tx | 1 | The aim of this study was to develop a patient-specific simulation for mitral valve repair and provide a proof of concept of personalized medicine in a patient prospectively planned for mitral valve surgery. | Mitral valve and negative mould were printed in systole to capture the pathology when the valve closes. A patient-specific mitral valve silicone replica was casted and mounted in the simulator. All repair techniques could be performed in the simulator to choose the best repair strategy. As the valve was printed in systole, no special testing other than adjusting the coaptation area was required. Subsequently, the patient was operated, mitral valve pathology was validated and repair was successfully done as in the simulation. | 4 |
| 89 | Dankowski R, Baszko A, Sutherland M, et al. 3D heart model printing for preparation of percutaneous structural interventions: description of the technology and case report. Kardiol Pol. 2014;72(6):546-551. | Review/Other-Tx | 1 | We intend to provide a description of 3D printing as a clinically applicable heart modelling technology for the planning of percutaneous structural heart procedures as well as to report our first clinical use of a 3D printed patient-specific heart model in preparation for a percutaneous mitral annuloplasty using the Mitralign percutaneous annuloplasty system. | The technique of direct percutaneous mitral annuloplasty requires advancement of a guiding catheter through the aorta, into the left ventricle, and requires the positioning of the tip of the catheter between the papillary muscles in close proximity to the mitral annulus. The 3D heart model was used to create a procedural plan to optimise potential device implantation. The size of the deflectable guiding catheter was selected on the basis of the patient's heart model. Target locations for annulus crossing wires were evaluated pre-procedurally using the individual patient's 3D heart model. In addition, the ability to position the Bident Catheter at the appropriate locations under the mitral annulus as well as the manoeuvrability between the papillary muscles were analysed on the heart model, enabling safe completion of the procedure, which resulted in a significant reduction in mitral regurgitation. | 4 |
| 90 | Muraru D, Hahn RT, Soliman OI, Faletra FF, Basso C, Badano LP. 3-Dimensional Echocardiography in Imaging the Tricuspid Valve. JACC Cardiovasc Imaging. 2019;12(3):500-515. | Review/Other-Tx | NA | Purpose statement related to 3D printing not clearly stated | No results stated in abstract. | 4 |
| 91 | Muraru D, Veronesi F, Maddalozzo A, et al. 3D printing of normal and pathologic tricuspid valves from transthoracic 3D echocardiography data sets. Eur Heart J Cardiovasc Imaging. 2017;18(7):802-808. | Observational-Dx | 5 | To explore the feasibility of using transthoracic 3D echocardiography (3DTTE) data to generate 3D patient-specific models of tricuspid valve (TV). | Antero-posterior (4.2 ± 0.2 cm vs. 4.2 ± 0 cm), ML (3.7 ± 0.2 cm vs. 3.6 ± 0.1 cm), P (12.6 ± 0.2 cm vs. 12.7 ± 0.1 cm), H (11.2 ± 2.1 mm vs. 10.8 ± 2.1 mm) and V (3.0 ± 0.6 ml vs. 2.8 ± 1.4 ml) were similar (P = NS for all) when measured on the 3D data set and the printed model. The two sets of measurements were highly correlated (r = 0.991). The mean absolute error (2D - 3D) for AP, ML, P and tenting H was 0.7 ± 0.3 mm, indicating accuracy of the 3D model of <1 mm. | 3 |
| 92 | Wang DD, Lee JC, O'Neill BP, O'Neill WW. Multimodality Imaging of the Tricuspid Valve for Assessment and Guidance of Transcatheter Repair. Interv Cardiol Clin. 2018;7(3):379-386. | Review/Other-Tx | NA | Combinations of preprocedural electrocardiography-gated cardiac computed tomography (CT), 3D printing, and 3D TEE have demonstrated to be useful in the intraprocedural visualization and planning of tricuspid interventions. | No results stated in abstract. | 4 |
| 93 | Harb SC, Rodriguez LL, Svensson LG, et al. Pitfalls and Pearls for 3-Dimensional Printing of the Tricuspid Valve in the Procedural Planning of Percutaneous Transcatheter Therapies. JACC Cardiovasc Imaging. 2018;11(10):1531-1534. | Review/Other-Tx | 4 | Here, we first present the steps involved in building a right-heart model for printing based on 4D computed tomography, and then illustrate tailored imaging approaches to overcome technical challenges in imaging, incorporating 3D transthoracic echocardiography, and cardiac magnetic resonance: imaging in the context of pacing leads; suboptimal visualization of the right-sided structures; and severe kidney dysfunction precluding contrast administration. The 3D printed models were helpful for procedural simulation | No abstract available. | 4 |
| 94 | Navia JL, Kapadia S, Elgharably H, et al. First-in-Human Implantations of the NaviGate Bioprosthesis in a Severely Dilated Tricuspid Annulus and in a Failed Tricuspid Annuloplasty Ring. Circ Cardiovasc Interv. 2017;10(12). | Review/Other-Tx | 2 | 1. Preoperative sizing included a focused 4-dimensional computed tomography that was used to develop a 3-dimensional printing model of the right heart structures to simulate the implantation steps of the NaviGate device. 2. Preoperative planning included testing in a 3-dimensional printing model | No abstract available. | 4 |
| 95 | O'Neill B, Wang DD, Pantelic M, et al. Transcatheter caval valve implantation using multimodality imaging: roles of TEE, CT, and 3D printing. JACC Cardiovasc Imaging. 2015;8(2):221-225. | Review/Other-Tx | 1 | This iPIX illustrates 3-dimensional (3D) printing guided periprocedural, multimodality pictorial planning performed for a successful transcatheter caval valve implantation | No abstract available. | 4 |
| 96 | Valverde I, Sarnago F, Prieto R, Zunzunegui JL. Three-dimensional printing in vitro simulation of percutaneous pulmonary valve implantation in large right ventricular outflow tract. Eur Heart J. 2017;38(16):1262-1263. | Review/Other-Tx | 1 | We used three-dimensional printing technology to test and simulate the dual stent patient-specific PPVI on the catheterization table. | No abstract available. | 4 |
| 97 | Schievano S, Migliavacca F, Coats L, et al. Percutaneous pulmonary valve implantation based on rapid prototyping of right ventricular outflow tract and pulmonary trunk from MR data. Radiology. 2007;242(2):490-497. | Observational-Dx | 12 | To determine if magnetic resonance (MR) imaging data can be used to create rigid models that are accurate representations of the right ventricular outflow tract (RVOT) and pulmonary trunk anatomy and if such models can be used to refine the selection of patients for percutaneous pulmonary valve implantation (PPVI). | For four subjects, both cardiologists correctly determined, on the basis of MR image or three-dimensional (3D) RP model findings, that PPVI should not have been attempted. Two patients in whom PPVI was attempted were considered to be unsuitable for the procedure after balloon sizing, and in another two patients, implantation was unsuccessful because of device instability. For the four patients in whom PPVI was suitable and the four in whom it was unsuitable, observers 1 and 2 correctly determined suitability for PPVI in four and two patients, respectively, by using the MR images alone. Both observers correctly determined the suitability of five patients by using the 3D models alone. | 2 |
| 98 | Armillotta A, Bonhoeffer P, Dubini G, et al. Use of rapid prototyping models in the planning of percutaneous pulmonary valved stent implantation. Proc Inst Mech Eng H. 2007;221(4):407-416. | Review/Other-Tx | NA | The paper reports the results of an experimental investigation on the use of arterial models built by rapid prototyping techniques. | The use of anatomical models has allowed the cardiologist's confidence in patient selection, prosthesis fabrication, and final implantation to be significantly improved. | 4 |
| 99 | Knecht S, Brantner P, Cattin P, Tobler D, Kuhne M, Sticherling C. State-of-the-art multimodality approach to assist ablations in complex anatomies-From 3D printing to virtual reality. Pacing Clin Electrophysiol. 2019;42(1):101-103. | Review/Other-Tx | 1 | We present a comprehensive overview of the current state-of-the-art modalities available to plan and guide catheter ablation in an ACHD patient. In addition to the clinical assessment of the computed tomography and the integration of 3D reconstructions into the electroanatomical mapping system, 3D printing and virtual reality assessment showed its value in preprocedural planning of the intervention. | No results stated in abstract. | 4 |
| 100 | Lodzinski P, Balsam P, Peller M, Gawalko M, Opolski G, Grabowski M. Three-dimensional print facilitated ventricular tachycardia ablation in patient with corrected congenital heart disease. Cardiol J. 2017;24(5):584-585. | Review/Other-Tx | 1 | An understanding of the precise surgical anatomy in such cases is often challenging and modern high resolution imaging techniques coupled with 3-dimensional (3D) printing may allow better visualization of the complex anatomy and improve planning of operations through hands-on simulation of surgical and interventional procedures | No abstract available. | 4 |
| 101 | Bauch T, Vijayaraman P, Dandamudi G, Ellenbogen K. Three-Dimensional Printing for In Vivo Visualization of His Bundle Pacing Leads. Am J Cardiol. 2015;116(3):485-486. | Review/Other-Tx | NA | This review describes and illustrates the application, development and associated limitation of additive manufacturing in the field of cardiology by studying research papers on AM in medicine/cardiology. | AM creates an accurate three-dimensional anatomical model to explain, understand and prepare for complex medical procedures. A prior study of patient's 3D heart model can help doctors understand the anatomy of the individual patient, which may also be used create training modules for institutions and surgeons for medical training. | 4 |
| 102 | Seckeler MD, White SC, Klewer SE, Ott P. Transjugular Transseptal Approach for Left Ventricular Pacing Lead in an Adult With Criss-Cross Heart. JACC Clin Electrophysiol. 2019;5(8):998-999. | Review/Other-Tx | 1 | This report presents the case of a patient with complex, post-operative congenital heart disease who required an atypical approach for placement of a left ventricular pacing lead to establish biventricular pacing after developing pacemaker-mediated cardiomyopathy | No abstract available. | 4 |
| 103 | Liddy S, McQuade C, Walsh KP, Loo B, Buckley O. The Assessment of Cardiac Masses by Cardiac CT and CMR Including Pre-op 3D Reconstruction and Planning. Curr Cardiol Rep. 2019;21(9):103. | Review/Other-Tx | NA | The purpose of this review is to (1) review the recent evidence examining the use of CT and CMR in the assessment of a suspected cardiac mass, (2) summarize the typical imaging features of the most common cardiac masses, and (3) examine the latest developments in the use of three-dimensional reconstructions and models in the preoperative assessment of a cardiac mass. | No results stated in abstract. | 4 |
| 104 | Young PM, Foley TA, Araoz PA, Williamson EE. Computed Tomography Imaging of Cardiac Masses. Radiol Clin North Am. 2019;57(1):75-84. | Review/Other-Tx | NA | Advances in CT technology, such as dual-energy CT, dynamic perfusion imaging, and three-dimensional printing for preoperative planning, will increase the role of CT in assessment of cardiac masses. | No results stated in abstract. | 4 |
| 105 | Riggs KW, Dsouza G, Broderick JT, Moore RA, Morales DLS. 3D-printed models optimize preoperative planning for pediatric cardiac tumor debulking. Transl Pediatr. 2018;7(3):196-202. | Review/Other-Tx | 2 | 3D-printed cardiac tumor models were used to identify the spacial relationship between the tumors and coronary arteries as well as understand the depth and infiltration of the tumors. | Patient 1 had a cardiac tumor arising from the anterior surface of the right ventricle causing significant right ventricular outflow tract obstruction and involving the right and left coronary artery courses. Patient 2 had a cardiac tumor arising from the left ventricle and extending beyond the left atrium compressing the airway preventing extubation, and surrounding the left coronary artery system. In both patients, 3D-printed models were used to maximize debulking and avoid injury to the coronaries. | 4 |
| 106 | Golab A, Slojewski M, Brykczynski M, et al. Three-Dimensional Printing as an Interdisciplinary Communication Tool: Preparing for Removal of a Giant Renal Tumor and Atrium Neoplastic Mass. Heart Surg Forum. 2016;19(4):E185-186. | Review/Other-Tx | 1 | We used 3D printing to plan for a rare complicated surgery involving the removal of a renal tumor and neoplastic mass, which reached the heart atrium | No results stated in abstract. | 4 |
| 107 | Al Jabbari O, Abu Saleh WK, Patel AP, Igo SR, Reardon MJ. Use of three-dimensional models to assist in the resection of malignant cardiac tumors. J Card Surg. 2016;31(9):581-583. | Review/Other-Tx | 2 | We report two patients with large complex cardiac tumors where 3D technology was utilized to analyze the tumor size, location, and extension more precisely, allowing better preoperative planning and decision making. | No results stated in abstract. | 4 |
| 108 | Son KH, Kim KW, Ahn CB, et al. Surgical Planning by 3D Printing for Primary Cardiac Schwannoma Resection. Yonsei Med J. 2015;56(6):1735-1737. | Review/Other-Tx | 1 | To facilitate surgical planning, we used 3D printing. Using a printed heart model, we decided that tumor resection under cardiopulmonary bypass (CPB) through sternotomy would be technically feasible. | At surgery, a huge tumor in the interatrial septum was confirmed. By incision on the atrial roof between the aorta and SVC, tumor enucleation was performed successfully under CPB. Pathology revealed benign schwannoma. The patient was discharged without complication. | 4 |
| 109 | Schmauss D, Gerber N, Sodian R. Three-dimensional printing of models for surgical planning in patients with primary cardiac tumors. J Thorac Cardiovasc Surg. 2013;145(5):1407-1408. | Review/Other-Tx | 1 | We are therefore exploring the impact of using rapid-prototyping techniques for decision making, surgical planning, and intraoperative orientation for surgical treatment in patients with primary cardiac tumors. | No abstract available. | 4 |
| 110 | Jacobs S, Grunert R, Mohr FW, Falk V. 3D-Imaging of cardiac structures using 3D heart models for planning in heart surgery: a preliminary study. Interact Cardiovasc Thorac Surg. 2008;7(1):6-9. | Review/Other-Tx | 3 | The aim of the study was to create an anatomical correct 3D rapid prototyping model (RPT) for patients with complex heart disease and altered geometry of the atria or ventricles to facilitate planning and execution of the surgical procedure. | The patient individual 3D printed RPT-models were used to plan the resection of a left ventricular aneurysm and right ventricular tumor. The surgeon was able to identify risk structures, assess the ideal resection lines and determine the residual shape after a reconstructive procedure (LV remodelling, infiltrating tumor resection). Using a 3D-print of the LV-aneurysm, reshaping of the left ventricle ensuring sufficient LV volume was easily accomplished. | 4 |
| 111 | Yoo JS, Reddy YNV, Kim KH. Heart transplantation for dextrocardia: preoperative planning using 3D printing. Eur Heart J Cardiovasc Imaging. 2019. | Review/Other-Tx | 1 | We developed a 3D printing model of the aorta, pulmonary artery, vena cave, and heart based on 512-slice cardiac CT angiography to visualize the complex anatomy of heart and formulate a detailed preoperative plan | No abstract available. | 4 |
| 112 | Thaker R, Araujo-Gutierrez R, Marcos-Abdala HG, Agrawal T, Fida N, Kassi M. Innovative Modeling Techniques and 3D Printing in Patients with Left Ventricular Assist Devices: A Bridge from Bench to Clinical Practice. J Clin Med. 2019;8(5). | Review/Other-Tx | NA | In this review article, we present some innovative modeling techniques that are often used in device development or for research purposes, but have not been utilized clinically | No results stated in abstract. | 4 |
| 113 | Miller J, Billadello J, Simon-Lee R, et al. 3D PRINTING FOR PREOPERATIVE PLANNING AND SURGICAL SIMULATION OF VAD IMPLANTATION IN A FAILING RIGHT SYSTEMIC VENTRICLE. Journal of the American College of Cardiology. 2018;71(11 Supplement):A545. | Review/Other-Tx | 1 | We aimed to determine the utility of two 3D printed models for preoperative planning by simulating VAD implantation. | Initially, the model identified where the aorta was adherent to the chest wall, preventing a potentially devastating complication. The multicolor model identified dense trabeculations requiring resection. Using this, a suitable place for the inflow cannula was determined. When correlated with the full-thorax model, it was found that this location would not impinge upon the diaphragm or ribs. An atriotomy was performed, allowing for a more complete evaluation than possible with the patient, which identified additional trabeculations requiring resection. Overall, both surgeons reported the models beneficial for surgical simulation and operative planning. Despite the excellent performance, 3 limitations were discovered. First, valve leaflets could not be printed. Second, in pre-simulation model manipulation, a tear in the ascending aorta occurred due to it being tethered. Lastly, the aorta was transected by a side-biting clamp. | 4 |
| 114 | Farooqi KM, Saeed O, Zaidi A, et al. 3D Printing to Guide Ventricular Assist Device Placement in Adults With Congenital Heart Disease and Heart Failure. JACC Heart Fail. 2016;4(4):301-311. | Review/Other-Tx | NA | hree-dimensional printing offers individualized structural models that would enable pre-surgical planning of cannula and device placement in adults with congenital cardiac disease and heart failure who are candidates for such therapies. We present a review of relevant cardiac anomalies, cases in which such models could be utilized, and some background on the cost and procedure associated with this process. | No results stated in abstract. | 4 |
| 115 | Fan Y, Kwok KW, Zhang Y, Cheung GS, Chan AK, Lee AP. Three-Dimensional Printing for Planning Occlusion Procedure for a Double-Lobed Left Atrial Appendage. Circ Cardiovasc Interv. 2016;9(3):e003561. | Review/Other-Tx | 1 | For better planning, we decided to simulate the actual procedure using three-dimensional (3D) printing technology | No abstract available. | 4 |
| 116 | Fan Y, Yang F, Cheung GS, et al. Device Sizing Guided by Echocardiography-Based Three-Dimensional Printing Is Associated with Superior Outcome after Percutaneous Left Atrial Appendage Occlusion. J Am Soc Echocardiogr. 2019;32(6):708-719 e701. | Observational-Tx | 32 | The aims of this study were to assess the association of model-based device selection with procedural safety and efficacy and to determine if preprocedural model testing leads to superior outcomes. | Patients in the retrospective cohort with model-mismatch sizing had longer procedure times, more implantation failures, more devices used per procedure, more procedural complications, more peridevice leak, more device thrombus, and higher cumulative incidence rates of ischemic stroke and cardiovascular or unexplained death (P < .05 for all) over 3.0 ± 2.3 years after LAA occlusion. Compared with the retrospective imaging-guided cohort, the prospective model-guided patients achieved higher implantation success and shorter procedural times (P < .05) without complications. Clinical device compression (r = 0.92) and protrusion (r = 0.95) agreed highly with model testing (P < .0001). Predictors for sizing mismatch were nonwindsock morphology (odds ratio, 4.7) and prominent LAA trabeculations (odds ratio, 7.1). | 2 |
| 117 | Morcos R, Al Taii H, Bansal P, et al. Accuracy of Commonly-Used Imaging Modalities in Assessing Left Atrial Appendage for Interventional Closure: Review Article. J Clin Med. 2018;7(11). | Review/Other-Tx | NA | We therefore sought to examine the accuracy of the most commonly utilized imaging modalities in LAA occlusion. | There is strong evidence that real-time three-dimensional transesophageal echocardiography is more accurate than two-dimensional transesophageal echocardiography. Three-dimensional computed tomography has recently emerged as an imaging modality and it showed exceptional accuracy when merged with three-dimensional printing technology. However, real time three-dimensional transesophageal echocardiography may be considered the preferred imaging modality as it can provide accurate measurements without requiring radiation exposure or contrast administration. | 4 |
| 118 | Hell MM, Achenbach S, Yoo IS, et al. 3D printing for sizing left atrial appendage closure device: head-to-head comparison with computed tomography and transoesophageal echocardiography. EuroIntervention. 2017;13(10):1234-1241. | Observational-Tx | 22 | We investigated whether the use of 3D-printed left atrial appendage (LAA) models based on preprocedural computed tomography (CT) permits accurate device sizing. | Implantation was successful in all patients. Mean LAA ostium diameter based on TEE was 22±4 mm and based on CT 25±3 mm (p=0.014). Predicted device size based on simulated implantation in the 3D model was equal to the device finally implanted in 21/22 patients (95%). TEE would have undersized the device in 10/22 patients (45%). Device compression determined in the 3D-CT model corresponded closely with compression upon implantation (16±3% vs. 18±5%, r=0.622, p=0.003). | 3 |
| 119 | Li H, Qingyao, Bingshen, et al. Application of 3D printing technology to left atrial appendage occlusion. Int J Cardiol. 2017;231:258-263. | Experimental-Tx | 21 | We assessed the feasibility and effectiveness of the 3DP technology for left atrial appendage (LAA). | All patients underwent a successful LAA occlusion operation with the Watchman device. TOE, LAA angiography, and cardiac computed tomography angiography measurements of the LAA orifice size between the groups were 20.4±2.5 vs. 20.1±3.3mm, 19.6±2.2 vs. 19.5±2.8mm, and 20.8±2.1 vs. 20.2±3.0mm, respectively (p>0.05). After the occlusion, the immediate TOE examination showed 3 mild residual shunt cases in the control group. The radiation exposure was significantly reduced in the 3DP compared with the control group (p<0.05). The patients were followed for an average of 7.7±2.5months. No postoperative complications, device-related thrombosis, or ischemic events occurred. | 2 |
| 120 | Goitein O, Fink N, Guetta V, et al. Printed MDCT 3D models for prediction of left atrial appendage (LAA) occluder device size: a feasibility study. EuroIntervention. 2017;13(9):e1076-e1079. | Observational-Dx and Tx | 29 | We assessed the feasibility of MDCT-based models to predict the correct size of device for LAA occlusion procedures. | Two procedures were aborted due to failure of occlusion; all three physicians predicted it. There was good correlation between the 3D models and the inserted device for AMPLATZER devices with a concordance correlation coefficient of 0.778 (p=0.001) and poor agreement for WATCHMAN devices - concordance correlation coefficient of 0.315 (p=0.203). Agreement among the three physicians for AMPLATZER and WATCHMAN devices was excellent, with a calculated average intra-class correlation of 0.915 and 0.816, respectively. | 2 |
| 121 | Conti M, Marconi S, Muscogiuri G, et al. Left atrial appendage closure guided by 3D computed tomography printing technology: A case control study. J Cardiovasc Comput Tomogr. 2018. | Observational-Dx and Tx | 20 | We sought to evaluate the additional value of left atrial appendage (LAA) 3D printing derived from computed tomography (CCT) in determining the size for LAA occlusion (LAAO) devices as compared to standard measurement by using occurrence of LAA leak as endpoint. | Compared to the 3D printed model, 55% of the devices were underestimated, the two sizing approaches agreed in 35% of the patients, while the 3D printed model overestimated the size in 10% of patients. The prevalence of LAA leak was significantly higher in the subset of patients with underestimation of prosthesis implanted with the standard approach as compared to the other patients (p = 0.019). | 3 |
| 122 | Litwinowicz R, Witowski J, Sitkowski M, et al. Applications of low-cost 3D printing in left atrial appendage closure using epicardial approaches - initial clinical experience. Kardiochir Torakochirurgia Pol. 2018;15(2):135-140. | Review/Other-Tx | 2 | We report as a first 2 cases of LAA occlusion procedure using 2 different systems: thoracoscopic AtriClip and the LARIAT device in which a 3D printed LAA model was used in preoperative planning. | In the first patient, preoperative measurements of 3D LAA model were performed using a dedicated selection guide for AtriClip device were comparable with the intraoperative examination. Left atrial appendage was closed epicardial using 40 mm size AtriClip. In second patients, LAA closure was performed completely percutaneously using LARIAT device. For better visualization of LAA shape on fluoroscopy and TEE examination, intraoperatively sterilized 3D LAA model was used during the procedure. In both cases, intraoperative TEE examination confirmed complete LAA closure with no leak. | 4 |
| 123 | Hachulla AL, Noble S, Guglielmi G, Agulleiro D, Muller H, Vallee JP. 3D-printed heart model to guide LAA closure: useful in clinical practice? Eur Radiol. 2019;29(1):251-258. | Observational-Tx | 15 | The aim of our study was to investigative the utility of personalized 3D-printed models (P3DPM) of the LAA to guide device size selection. | The device size predicted by 3D-TEE and CT corresponded to the implanted device size in 8/15 (53%) and 10/15 (67%), respectively. The predicted device size from the P3DPM was accurate in all patients, obtaining perfect contact with the LAA wall, without device instability or excessive compression. P3DPM-CT with the deployed device showed device deformation and positioning of the disk in relation to the pulmonary veins, allowing us to determine the best device size in all 15 cases. | 2 |
| 124 | Iriart X, Ciobotaru V, Martin C, et al. Role of cardiac imaging and three-dimensional printing in percutaneous appendage closure. Arch Cardiovasc Dis. 2018;111(6-7):411-420. | Review/Other-Tx | NA | These imaging modalities, including transoesophageal echocardiography and multislice computed tomography, allow acquisition of a three-dimensional dataset that improves understanding of the cardiac anatomy; dedicated postprocessing software integrated into the clinical workflow can be used to generate a stereolithography file, which can be printed in a rubber-like material, seeking to replicate the myocardial tissue characteristics and mechanical properties of the left atrial appendage wall. | No results stated in abstract. | 4 |
| 125 | Ciobotaru V, Combes N, Martin CA, et al. Left atrial appendage occlusion simulation based on three-dimensional printing: new insights into outcome and technique. EuroIntervention. 2018;14(2):176-184. | Observational-Dx and Tx | 76 | The aim of this study was to assess the predictive value of simulation based on 3D-printed models before left atrial appendage occlusion (LAAO) for peri-device leaks (PDL) and the impact on procedural outcomes compared to conventional imaging. | In Group 1, an off-axis device position occurred in 14 patients (25%) and the incidence of PDL was 27% (15 patients); mismatch between model and device size was the best predictor (area under the curve 0.88, CI: 0.77-0.99). When using 3D simulation prospectively, mean prosthesis number per patient (1.05±0.21 vs. 1.20±0.52, p=0.04) and incidence of leaks (5% vs. 27%, p<0.01) were reduced compared to conventional imaging alone, as well as fluoroscopy time (19 mins [13.4-23] vs. 13.5 mins [11.1-15], p=0.012) and total fluoroscopy dose (7,291 [1,811-12,734] vs. 1,978 (1,548-4,800) mGy·cm2, p=0.029). | 2 |
| 126 | Wang DD, Eng M, Kupsky D, et al. Application of 3-Dimensional Computed Tomographic Image Guidance to WATCHMAN Implantation and Impact on Early Operator Learning Curve: Single-Center Experience. JACC Cardiovasc Interv. 2016;9(22):2329-2340. | Observational-Tx | 53 | The aim of this study was to examine the impact of 3-dimensional (3D) computed tomographic (CT) guided procedural planning for left atrial appendage (LAA) occlusion on the early operator WATCHMAN learning curve. | All 53 patients underwent successful LAA occlusion with the WATCHMAN. Three-dimensional CT LAA maximal-width sizing was 2.7 ± 2.2 mm and 2.3 ± 3.0 mm larger than 2-dimensional and 3D TEE measurements, respectively (p ≤ 0.0001). By CT imaging, device selection was 100% accurate. There were 4 peri-WATCHMAN leaks (<4.5 mm) secondary to accessory LAA pedunculations. By 2-dimensional TEE maximal-width measurements alone, 62.3% (33 of 53) would have required larger devices. Using 3D TEE maximal-width measurements, 52.8% of cases (28 of 53) would have required larger devices. Three-dimensional TEE length would have inappropriately excluded 10 patients from WATCHMAN implantation. Compared with the average of 1.8 devices used per implantation attempt in PROTECT AF (WATCHMAN Left Atrial Appendage System for Embolic Protection in Patients With Atrial Fibrillation) (82% success rate), the present site averaged 1.245 devices per implantation attempt (100% success rate). There were no intraprocedural screen failures and no major adverse cardiac events. | 2 |
| 127 | Obasare E, Mainigi SK, Morris DL, et al. CT based 3D printing is superior to transesophageal echocardiography for pre-procedure planning in left atrial appendage device closure. Int J Cardiovasc Imaging. 2018;34(5):821-831. | Observational-Dx and Tx | 14 | Sizing is traditionally done with transesophageal echocardiography (TEE) but this is not always precise. Three-dimensional (3D) printing of the LAA may be more accurate. | The model correlated perfectly with implanted device size (R2 = 1; p < 0.001), while TEE-predicted size showed inferior correlation (R2 = 0.34; 95% CI 0.23-0.98, p = 0.03). Fisher's exact test showed the model better predicted final WD size than TEE (100 vs. 60%, p = 0.02). Use of the model was associated with reduced procedure time (70 ± 20 vs. 107 ± 53 min, p = 0.03), anesthesia time (134 ± 31 vs. 182 ± 61 min, p = 0.03), and fluoroscopy time (11 ± 4 vs. 20 ± 13 min, p = 0.02). Absence of peri-device leak was also more likely when the model was used (92 vs. 56%, p = 0.04). There were trends towards reduced trans-septal puncture to catheter removal time (50 ± 20 vs. 73 ± 36 min, p = 0.07), number of device deployments (1.3 ± 0.5 vs. 2.0 ± 1.2, p = 0.08), and number of devices used (1.3 ± 0.5 vs. 1.9 ± 0.9, p = 0.07). | 2 |
| 128 | Khalili H, Gentry RE, Stevens MA, et al. Rapid and Affordable 3-Dimensional Prototyping for Left Atrial Appendage Closure Planning. Circ Cardiovasc Interv. 2017;10(2):e004710. | Review/Other-Tx | 1 | Multiple Watchman device sizes were tested in the printed model for the best fit | No abstract available. | 4 |
| 129 | Liu P, Liu R, Zhang Y, Liu Y, Tang X, Cheng Y. The Value of 3D Printing Models of Left Atrial Appendage Using Real-Time 3D Transesophageal Echocardiographic Data in Left Atrial Appendage Occlusion: Applications toward an Era of Truly Personalized Medicine. Cardiology. 2016;135(4):255-261. | Observational-Dx | 8 | The objective of this study was to assess the clinical feasibility of generating 3D printing models of left atrial appendage (LAA) using real-time 3D transesophageal echocardiogram (TEE) data for preoperative reference of LAA occlusion. | We successfully printed LAAs of 8 patients. Each LAA costs approximately CNY 800-1,000 and the total process takes 16-17 h. Seven of the 8 Watchman devices predicted by preprocedural 2D TEE images were of the same sizes as those placed in the real operation. Interestingly, 3D printing models were highly reflective of the shape and size of LAAs, and all device sizes predicted by the 3D printing model were fully consistent with those placed in the real operation. Also, the 3D printed model could predict operating difficulty and the presence of a peridevice leak. | 3 |
| 130 | Pellegrino PL, Fassini G, M DIB, Tondo C. Left Atrial Appendage Closure Guided by 3D Printed Cardiac Reconstruction: Emerging Directions and Future Trends. J Cardiovasc Electrophysiol. 2016;27(6):768-771. | Review/Other-Tx | 2 | 3D printing is a novel technology able to create a patient specific model of any given anatomical portion of the heart. | Herein we report the first 2 cases of LAA occlusion procedure with 2 different systems, the Wave Crest device (Coherex Medical, Inc., USA) and the Amplatzer Amulet device (St. Jude Medical, St. Paul, MN, USA), in which a 3D printed LAA model (Care Tronik, Prato, Italy) was used in a rehearse phase. Both patients had history of paroxysmal AF and previous transient ischemic attack (TIA) occurred during oral anticoagulation with correct INR. In the first patient the occlusive device was positioned within the LAA after a rehearse occlusion using the 3D printed LAA plus a 27 mm Coherex Wavecrest device, demonstrating a good compression and sealing, particularly considering a proximal lobe of the appendage. In the second patient an attempt with the 27 mm Amulet device delivered within the 3D printed LAA, based on angiography and transesophageal echocardiographic (TEE), revealed insufficient covering of the proximal part of LAA vestibule; the device was released only after a second test with the 31 mm Amulet demonstrating a good sealing. | 4 |
| 131 | Otton JM, Spina R, Sulas R, et al. Left Atrial Appendage Closure Guided by Personalized 3D-Printed Cardiac Reconstruction. JACC Cardiovasc Interv. 2015;8(7):1004-1006. | Review/Other-Tx | 1 | The imaged 3D printed replica atrial appendage with the devices in situ was analyzed (3-Matic 9.0, Materialise Software), and the anatomic deformation was calculated for each device, creating a 3D map color-coded according to the degree of deformation caused. This demonstrated the areas and extent of engagement of the device on the flexible atrial model. | No abstract available. | 4 |
| 132 | Song H, Zhou Q, Zhang L, et al. Evaluating the morphology of the left atrial appendage by a transesophageal echocardiographic 3-dimensional printed model. Medicine (Baltimore). 2017;96(38):e7865. | Review/Other-Tx | 18/2 used for rehearsal | This study aimed to determine the feasibility of 3D-printed left atrial appendage (LAA) models based on 3D transesophageal echocardiography (3D TEE) data and their application value in treating LAA occlusions | 3D prints exhibited excellent cardiac imaging and planning of the 'optimal' volume and shape of the resection, as well as intraoperative quality control of septal myectomy by filling a trough in the 3D-printed interventricular septum with excised myocardium. | 4 |
| 133 | Andrushchuk U, Adzintsou V, Nevyglas A, Model H. Virtual and real septal myectomy using 3-dimensional printed models. Interact Cardiovasc Thorac Surg. 2018;26(5):881-882. | Review/Other-Tx | 2 | We present 2 cases of extended septal myectomy using low-cost 3-dimensional (3D) printed models of the interventricular septum with its fragment cut out mechanically or by initial 'virtual' myectomy. |  | 4 |
| 134 | Andrushchuk U, Adzintsou V, Niavyhlas A, Model H, Ostrovsky Y. Early results of optimal septal myectomy using 3-dimensional printed models. Kardiochir Torakochirurgia Pol. 2019;16(2):74-80. | Observational-Dx and Tx | 30 | To improve results in centres treating few patients with HOCM using a new method of optimal SM with the help of 3-dimensional models to achieve an 'ideal' interventricular septum (IVS) thickness of 10-11 mm. | Initial isolated extended SM (n = 29, 97%) was effective in 23/29 (79%) patients. Four non-fatal complications were observed. A permanent pacemaker was implanted in three patients. No patients required mitral valve replacement. The mean postoperative left ventricle (LV) resting systolic gradient was 7.5 ±4.4 mm Hg, and at the latest follow-up this value was 7.1 ±4.2 mm Hg. The average weight of the excised myocardium was 12.0 g (range: 5.8-22.5 g). At follow-up both volumetric and dimensional LV echocardiography parameters increased compared with preoperative values (p ≤ 0.007). | 2 |
| 135 | Sun X, Zhang H, Zhu K, Wang C. Curved section modeling-based three-dimensional printing for guiding septal myectomy. J Thorac Dis. 2018;10(7):E535-E537. | Review/Other-Tx | 1 | Here, we investigated the curved section modeling technique from surgeon’s view angle for 3D printing of HOCM model. | No abstract available. | 4 |
| 136 | Hermsen JL, Yang R, Burke TM, et al. Development of a 3-D printing-based cardiac surgical simulation curriculum to teach septal myectomy. J Thorac Cardiovasc Surg. 2018;156(3):1139-1148 e1133. | Review/Other-Tx | 5 | We sought to develop a 3-D printing-based simulator for teaching extended septal myectomy to trainees in cardiothoracic surgery (clinical postgraduate year 4-7). | Baseline myectomy resection volumes differed significantly (attending 15 cm3 vs resident 3.1 cm3; P < .05). Residents resected increasingly larger volumes of tissue over the course of the study. Initial resection volume (compared with faculty) increased by 0.82 cm3 per resection (95% confidence interval, 0.37-1.3 cm3; P < .0001). Total resection volume (compared with faculty) increased by 3.6 cm3 per resection (95% confidence interval, 2.4-4.9 cm3; P < .0001). The residents' survey assessment of the simulator was favorable. | 4 |
| 137 | Guo HC, Wang Y, Dai J, Ren CW, Li JH, Lai YQ. Application of 3D printing in the surgical planning of hypertrophic obstructive cardiomyopathy and physician-patient communication: a preliminary study. J Thorac Dis. 2018;10(2):867-873. | Observational-Dx and Tx | 7 | The aim of this study was to evaluate the effect of 3-dimensional (3D) printing in treatment of hypertrophic obstructive cardiomyopathy (HOCM) and its roles in doctor-patient communication. | The heart anatomies were accurately printed with 3D technology. The 3D-printed prototypes were useful for preoperative evaluation, surgical planning, and practice. Preoperative and postoperative echocardiographic evaluation showed left ventricular outflow tract (LVOT) obstruction was adequately relieved (82.71±31.63 to 14.91±6.89 mmHg, P<0.001), the septal thickness was reduced from 21.57±4.65 to 17.42±5.88 mm (P<0.001), and the SAM disappeared completely after the operation. Patients highly appreciated the role of 3D model in preoperative conversations and the communication score was 9.11±0.38 points. | 4 |
| 138 | Hamatani Y, Amaki M, Kanzaki H, et al. Contrast-enhanced computed tomography with myocardial three-dimensional printing can guide treatment in symptomatic hypertrophic obstructive cardiomyopathy. ESC Heart Fail. 2017;4(4):665-669. | Review/Other-Tx | 1 | Here, we presented a young female patient with drug-refractory symptomatic HOCM. In this case, contrast-enhanced computed tomography enabled us to assess the suitability of percutaneous transluminal septal myocardial ablation. | By creating three-dimensional printed models using computed tomography data, we could also visualize intracardiac structure and simulate the surgical procedure. | 4 |
| 139 | Parachuri VR, Adhyapak SM. The case for surgical myectomy in hypertrophic cardiomyopathy: Is strategic planning the key to success? J Thorac Cardiovasc Surg. 2017;154(5):1687-1688. | Review/Other-Tx | NA | Comment and suggestion on another study | No abstract available. | 4 |
| 140 | Yang DH, Kang JW, Kim N, Song JK, Lee JW, Lim TH. Myocardial 3-Dimensional Printing for Septal Myectomy Guidance in a Patient With Obstructive Hypertrophic Cardiomyopathy. Circulation. 2015;132(4):300-301. | Review/Other-Tx | 1 | A CT 3-chamber view and a color-coded myocardial thickness map generated by CT data showed asymmetrical thickening of the LV myocardium that predominantly involved the ventricular septum and had a maximal thickness of 26 mm. | No abstract available. | 4 |
| 141 | Johnston NF, Prendiville T, McMahon CJ. 3D printing of severe hypertrophic cardiomyopathy in a child with Rasopathy. Ir J Med Sci. 2018;187(1):55-57. | Review/Other-Tx | 1 | We describe the use of 3D printing in conjunction with echocardiography in assessing hypertrophic cardiomyopathy in a boy with Rasopathy. | No abstract available. | 4 |
| 142 | Veselka J, Adla T, Adlova R, Bartel T. Three-Dimensional Heart Printing for Planning of Septal Reduction Therapy in Patients with Hypertrophic Obstructive Cardiomyopathy. Int J Angiol. 2018;27(3):165-166. | Review/Other-Tx | Not clearly stasted | For better visualization of left ventricular pathology and to improve decision making about a type of septal reduction, a three-dimensional printing of the heart might be performed using data from cardiac computed tomography (CT) examination. | No abstract available. | 4 |
| 143 | Hermsen JL, Burke TM, Seslar SP, et al. Scan, plan, print, practice, perform: Development and use of a patient-specific 3-dimensional printed model in adult cardiac surgery. J Thorac Cardiovasc Surg. 2017;153(1):132-140. | Review/Other-Tx | 2 | Static 3-dimensional printing is used for operative planning in cases that involve difficult anatomy. An interactive 3D print allowing deliberate surgical practice would represent an advance. | There was congruence between volumes of print and patient resection specimens (patient 1, 3.5 cm3 and 3.0 cm3, respectively; patient 2, 4.0 cm3 and 4.0 cm3, respectively). The prints were rated useful (3.5 and 3.6 on a 5-point Likert scale) for preoperative visualization, planning, and practice. Intraoperative echocardiographic assessment showed adequate relief of left ventricular outflow tract obstruction (patient 1, 80 mm Hg to 18 mm Hg; patient 2, 96 mm Hg to 9 mm Hg). Both patients reported symptomatic improvement (New York Heart Association functional class III to class I). | 4 |
